# Novelty selectively permits learning-associated plasticity in ventral tegmental-hippocampal-prefrontal circuitry

**DOI:** 10.3389/fnbeh.2022.1091082

**Published:** 2023-01-09

**Authors:** Alan Jung Park

**Affiliations:** ^1^Department of Physiology, Seoul National University College of Medicine, Seoul, Republic of Korea; ^2^Neuroscience Research Institute, Seoul National University College of Medicine, Seoul, Republic of Korea; ^3^The Mortimer B. Zuckerman Mind Brain Behavior Institute at Columbia University, New York, NY, United States

**Keywords:** novelty, dopamine D1-receptor, learning, theta, dopamine

## Abstract

Modifying established behavior in novel situations is essential, and patients with neuropsychiatric disorders often lack this flexibility. Understanding how novelty affects behavioral flexibility therefore has therapeutic potential. Here, novelty differentially impacts connectivity within the ventral tegmental-hippocampal-medial prefrontal (VTA-HPC-mPFC) circuit, thereby enhancing the ability of mice to overcome established behavioral bias and adapt to new rules. Circuit connectivity was measured by local field potential (LFP) coherence. As mice exposed to novelty learned to overcome previously established spatial bias, the ventral HPC (vHPC) strengthens its coherence with the VTA and mPFC in theta frequency (4–8 Hz). Novelty or learning did not affect circuits involving the dorsal HPC (dHPC). Without novelty, however, mice continued following established spatial bias and connectivity strength remained stable in the VTA-HPC-mPFC circuit. Pharmacologically blocking dopamine D1-receptors (D1Rs) in the vHPC abolished the behavioral and physiological impacts of novelty. Thus, novelty promotes behavioral adaptation by permitting learning-associated plasticity in the vHPC-mPFC and VTA-vHPC circuit, a process mediated by D1Rs in the vHPC.

## 1. Introduction

The ability to cope with novel situations is critical for everyday life, and this cognitive flexibility is impaired in patients with neuropsychiatric disorders including autism and schizophrenia (Geurts et al., 2009; Waltz, 2017). Although human and animal studies have shown that novelty exposure facilitates memory retention and can prevent dementia (Moncada and Viola, 2007; Fenker et al., 2008; Ballarini et al., 2009; Fissler et al., 2013), little is known about the circuit-level underpinnings of novelty effects on the acquisition of relevant information for behavioral adaptation. It has been shown that novelty recruits a wide range of brain regions including the mPFC, vHPC, and VTA, the major dopamine source for the vHPC (Yamaguchi, 2004; Fenker et al., 2008; Otmakhova et al., 2013; Bunzeck and Thiel, 2015). These findings suggest that novelty modulates specific brain circuits for the effective processing of incoming information. Understanding how these novelty-associated circuits interact for better learning and memory may lead to therapeutic interventions for cognitive impairments associated with neuropsychiatric disorders.

A recent study demonstrated that novelty exposure facilitates learning new rules, overcoming established old behavioral strategies. By measuring the phase-locking of mPFC single neuronal spiking to vHPC theta oscillations, they found that novelty weakens vHPC-mPFC connectivity that was established from previous experience. When mice are learning new rules, this weakened theta connectivity re-strengthens as the circuit encodes information about the new rule (Park et al., 2021). This suggests that novelty enhances behavioral flexibility by permitting learning-associated plasticity in the vHPC-mPFC circuit in theta frequency. However, maintaining strong theta connectivity prevents this plasticity and learning (Park et al., 2021). Indeed, a series of studies shows that aberrantly strong rigid connectivity within the hippocampal-prefrontal circuit is linked to increased perseverance to a previously established behavioral strategy, impairing adaptation to new task rules (Latif-Hernandez et al., 2016; Shah et al., 2018; Park et al., 2021). Therefore, it is important to determine whether novelty exerts its action by promoting plasticity in theta frequency in brain circuitry.

The VTA provides dopaminergic inputs to the vHPC, dHPC, and mPFC (Kempadoo et al., 2016; Morrens et al., 2020; Park et al., 2021). Recent findings suggest that dopaminergic inputs from the VTA may modulate vHPC activities in response to novelty *via* D1Rs, which in turn affects vHPC-mPFC connectivity (Park et al., 2021). Moreover, dopaminergic inputs from the VTA to the mPFC enhance the learning rate after novelty and gates vHPC-mPFC connectivity through D1Rs (Gurden et al., 1999, 2000; Morrens et al., 2020). Also, inhibiting D1Rs in the dHPC impairs novelty-induced enhancement of memory retention (Lemon and Manahan-Vaughan, 2006). These findings indicate that novelty tightly modulates the brain circuit composed of the VTA, vHPC, dHPC, and mPFC. However, how novelty affects plasticity in this circuit through learning has yet to be investigated.

We hypothesized that novelty facilitates behavioral flexibility by differentially modulating brain circuitry composed of the VTA, vHPC, dHPC, and mPFC in theta frequency. To test this hypothesis, we performed simultaneous *in vivo* local field potential (LFP) recordings in these areas. Analyzing LFP coherence between these regions revealed that novelty permits learning-associated plasticity specifically in the vHPC-mPFC, and VTA-vHPC circuits in theta frequency. These effects are mediated by D1Rs in the vHPC.

## 2. Materials and methods

This study used a subset of data that were collected for Park et al. (2021). Specifically, data from the mice implanted with LFP wires in the vHPC and VTA (see section “2.2.1 Drive implant”) were re-visited. All mice were males. Of note, VTA recordings were not reported in Park et al. (2021).

### 2.1. Subjects

Three-month-old male C57BL/6J mice (Jackson Labs) were group-housed on a 12-h light/12-h dark cycle with lights on at 7 am. Food and water were available *ad libitum*. After chronic drive/cannula implants, a pair of mice were housed in each compartment of a cage divided in half by a perforated plastic divider. Mice were randomly assigned to each experimental group, and behavioral experiments were conducted between 9 am and 7 pm. All procedures followed the NIH Guidelines and were approved by Columbia University and the New York State Psychiatric Institute Institutional Animal Care and Use Committees (IACUC).

### 2.2. Surgical procedures

Mice were placed in a flow box and exposed to 2% isoflurane. Once sedated, mice were placed in a stereotaxic apparatus, and dexamethasone (0.05 mL) and carprofen (0.15 mL) were administered subcutaneously. Isoflurane was maintained at 0.8% during the surgery, and a heating pad was used to maintain body temperature. Behavioral experiments were performed 4 weeks after the surgery, once the mice recovered.

#### 2.2.1. Drive implant

A 76-μm-diameter tungsten wire field electrode was implanted into the vHPC (ventral CA1/subiculum; 3.2 mm posterior to, 3.3 mm lateral to, 4.59 mm below bregma), dHPC (CA1 pyramidal layer; 1.9 mm posterior to, 1.3 mm lateral to, 1.26 mm below bregma), and VTA (3.2 mm posterior to, 0.32 mm lateral to, 4.43 mm below bregma). A bundle of 13 tungsten wire stereotrodes (13 μm diameter) was implanted into the mPFC (prelimbic/infralimbic cortex, layer II/III;1.8 mm anterior to, 0.3 mm lateral to, 2.1 mm below bregma) (Supplementary Figure 1). A reference screw was placed in the skull above the frontal cortex, and a ground screw was installed in the skull above the cerebellum. A 36-channel electrode interface board (Neuralynx) was dental cemented to the skull and then the wires were connected to it.

#### 2.2.2. Cannula implant

For drug infusion, guide cannulae (26 gauge; Plastics One, Roanoke, VA, USA 24018) were implanted into the vHPC (bilateral, angled by 10 degrees, 3.2 mm posterior to, 3.88 mm lateral to, 3.3 mm below bregma). The dummy cannulae (Plastics One) were inserted into the guide cannulae to prevent clogging. For drug infusion, the dummy cannulae were replaced by 33-gauge internal cannulae with a 0.5 mm projection. For LFP recordings in the vHPC, a tungsten wire field electrode was attached to a guide cannula with a 0.7 mm projection (Supplementary Figure 1).

### 2.3. Histology

To verify electrode placements, mice were anesthetized with a ketamine/xylazine mix, and a 50 μA current was passed through an electrode for 20 s to make lesions at the tip of the recording electrode. Mice were perfused first transcardially with PBS, and then with 4% paraformaldehyde in PBS. Brains were collected and fixed overnight in 4% paraformaldehyde at 4°C. Cryoprotection was performed at 4°C in 30% phosphate-buffered sucrose for 3 days. Brain sections (40 μm) were made using a cryostat and mounted. DAPI Fluoromount-G mounting medium (Southern Biotech, Cat. #: 0100-20) was used. Analyses were conducted on recordings only from verified recording sites.

### 2.4. Drug

A 100 mM stock solution of the standard selective D1-like antagonist SCH23390 (Tocris, Cat. #: 0925) was prepared in saline. On an experimental day, it was prepared at 1 mM final concentration in saline.

### 2.5. Behavior

Mice were allowed to recover for 4 weeks after surgeries. Mice were food restricted starting 3 days before the onset of free choice sessions until the end of behavioral experiments and maintained at 85% of the pre-restriction weight. Mice were gently handled for 3 days (3 min per day) before commencing behavioral experiments so that the mice were acclimated to experimenters.

The dimension of the circular arena was 50 cm in diameter and 25 cm high. Each arm of the custom-built automated T-maze was 10 cm wide, 32 cm long, and 15 cm high. The center arm was 55 cm long.

Novelty exposure evokes a mixture of behavioral states such as anxiety. Therefore, experiments were designed to minimize the non-specific effects of anxiety. It has been shown that exposing mice to an open arena under bright light elicits anxiety-related behavior and increases vHPC-mPFC synchrony. Notably, these effects were abolished when the same experiments were conducted in the dark (Adhikari et al., 2010). Another important observation is that delivering bright light abrogates novelty-induced synaptic depression (Manahan-Vaughan and Braunewell, 1999). Therefore, mice undertook all behavioral experiments in the dark to minimize the non-specific influence of anxiety. Indeed, the mice did not exhibit anxiety-related behavior during novelty exposure (reduced path length and time spent in the center of the arena) (Adhikari et al., 2010; Park et al., 2021).

**FIGURE 1:**
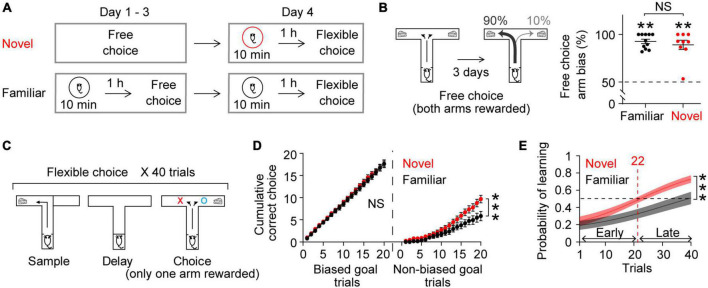
Novel experience enhances flexible learning. **(A)** Experimental design. **(B)** Mice established an arm bias after 3 days of free choice sessions, before exploring a novel (*n* = 9) or a familiar (*n* = 11) arena on day 4 (Wilcoxon signed-rank test, Novel, *P* = 0.004; Familiar, *P* = 0.001; Novel vs. Familiar, Mann Whitney test, *P* = 0.8). **(C)** Flexible choice task. The mice had to flexibly choose a goal side opposite to the randomly given sample goal. **(D)** Both groups of mice performed similarly when the choice goal matched their biased arm side [Left, trial × group, *F*_(19,342)_ = 0.3, *P* = 0.9]. However, the novel group displayed an improved performance at later trials when the choice goal mismatched their biased arm side [Right, trial × group: *F*_(19,342)_ = 4.4, *P* < 0.0001]. **(E)** Logistic regression model learning curve. Mice exposed to novelty progressively performed better in flexible choice training than those exposed to the familiar arena [trial × group: *F*_(39,702)_ = 2.7, *P* < 0.0001]. The average inflection point (learning trial) was 22 (Novel), and 44 (Familiar) (Mann–Whitney test, *P* = 0.02). Insets, learning trial of each mouse. The learning trial of two mice in the Familiar group was not defined because the overall slope of their learning curve was negative, indicating that learning had not occurred. Two-way RM ANOVA test for **(D,E)**. NS. not significant, **P < 0.005, ***P < 0.0005. Data are represented as mean ± SEM. **[B** (left), **C]** Adopted from Park et al. (2021).

In free choice, mice freely chose an arm to get a milk reward (diluted 1:3 in deionized water) in the T-maze. Once rewarded from one of the two goal arms, mice must return to and obtain a reward in the start box to initiate the next round of rewards. In flexible choice, mice underwent 40 trials of training in a delayed-non-match-to-sample paradigm in the same T-maze. The delayed-non-match-to-sample task has 3 phases: sample, delay, and choice. In the sample phase, mice were directed to a randomly selected goal arm to receive the reward. Then they returned to and remained in the start box for a 60-s delay. In the choice phase, the mice had to choose the goal arm opposite to the sample goal to successfully get the reward (Figure 1C). Therefore, depending on the randomly given sample goal, mice had to flexibly choose an arm side to get rewarded in the choice phase. On days 1–3, Mice in the familiar group explored the circular arena (30 min) and 1 h later underwent 30-min free choice sessions in the T-maze. Mice in the novel group underwent only free choice sessions. As a result, mice in the familiar group were familiarized with the circular arena. The circular arena and the T-maze were placed on a roller table each, and either apparatus was located in the same area at the time of behavioral experiments under the same behavior camera. On day 4, the novel and familiar groups explored the circular arena for 10 min and 1 h later underwent 40 trials of flexible choice training in the delayed-non-match-to-sample paradigm. As the reward rule had switched from free choice to flexible choice, mice had to overcome free choice behavior and adapt to the flexible choice task in order to obtain the reward. Of note, the novel group explored the circular arena for the very first time on day 4. There was a 1-h interval between arena exposure and flexible choice training because the effects of novelty generally last about 2 h (Moncada and Viola, 2007; Ballarini et al., 2009; Takeuchi et al., 2016).

It should be noted that the delayed-non-match-to-sample flexible choice task used in this study is fundamentally distinct from the widely used delayed-non-match-to-sample working memory task (Spellman et al., 2015; Abbas et al., 2018). In a conventional T-maze working memory task, mice are “shaped” for a few days to help mice spontaneously alternate goal arms. Then the mice undergo days of delayed non-match-to-sample training until they acquire the rule and perform over 70% correct levels, which is followed by working memory testing sessions. Therefore, the conventional T-maze working memory test examines the unbiased spatial working memory of mice that already know about the delayed non-match-to-sample rule (Spellman et al., 2015; Abbas et al., 2018). Notably, the flexible choice task does not have shaping as well as repeated training sessions. Consequently, mice developed a strong arm bias after free choice sessions, and they had to overcome this bias for successful performance in the delayed-non-match-to-sample flexible choice task. Importantly, the arm bias was not guided by external cues because (1) both goal arms were the same (color, scent, brightness, reward amount, etc.), (2) the experimental area was oriented consistently, and (3) mice were not biased exclusively to one side.

### 2.6. D1 antagonist experiment

SCH23390 (SCH, 100 nl, 1 mM) or vehicle (saline, 100 nl) was delivered bilaterally to the vHPC at 50 nl/min using a 10 μL Hamilton syringe and a Harvard Apparatus Pump II Dual Syringe micropump. After the injection, the injection cannulae were left in place for another 5 min to let the injected fluid diffuse. This treatment did not affect animal movement (Park et al., 2021), and the injected mice explored the novel circular arena 20 min later (Figure 3A). It has been shown that infusing 3.1 mM of SCH (as opposed to 1 mM in this study) into the dHPC impairs enhanced memory consolidation after novelty (Takeuchi et al., 2016). However, infusing this high concentration of SCH into the vHPC resulted in bradykinesia for a few hours. Notably, mice were acclimated to the cannulation procedures for 5 days prior to the drug injection to minimize the potential novelty of the cannulation procedures.

### 2.7. Learning curve

Learning curve during flexible choice training was examined using a logistic regression model. The MATLAB (MathWorks) function “glmft” was used to obtain estimates of logistic regression coefficients. These estimates were then fed into the “glmval” function to calculate predicted levels of learning performance. Because mice displayed strong arm bias (Figure 1B), there is a possibility that the performance of mice could be falsely considered as correct when the goal arm matched the biased arm. This potential error was countered by weighting logistic regression coefficients by the probability (P) of arm bias on the last day of the free choice session. If *P* > 0.7, correct trials when the goal arm matched the biased arm were weighted by 1—P. In case of no strong arm bias (*P* < 0.7), all correct observations regardless of arm sides were weighted by 1—P. This probability weighting was essential because mice outperformed when the goal arm matched the biased arm (Figure 1D). To estimate at which trial mice learned the flexible choice rule, the inflection point of the learning curve was determined. The curvature of the learning curve changes its direction at the inflection point, which reflects learning. For the two mice in the familiar group, their inflection points were not estimated because their learning curves were overall negative, indicating that mice did not learn.

### 2.8. Neural data analysis

LFP recordings were collected at 2 kHz while mice were performing during the flexible choice task. For mPFC LFP recordings, one electrode in the cc bundle was randomly selected. A Digital Lynx system (Neuralynx, Bozeman, MT, USA) was used to amplify, band-pass filter (1–1000 Hz), and digitize the recorded electrophysiological signals. Data analysis was conducted using custom-written scripts in MATLAB. Considering the influence of animal movement on LFP recordings (Buzsáki et al., 2012), LFP analyses were performed when mice were running in one direction at a comparable speed in the center arm, before turning to a goal arm (Supplementary Figure 2). To account for the non-specific effects of impedance differences across electrodes, raw LFP data were normalized by dividing the LFP signal, which was obtained when mice were running in the center arm in the choice phase, by the root mean square of the LFP signal over the whole recording session (MATLAB code rms was used). To calculate coherence, the wavelet method (the MATLAB wavelet toolbox) was used. Specifically, MATLAB code wcoherence with the sampling rate 2000 as an input parameter was used (Lowes et al., 2021).

### 2.9. Statistics

Statistical analysis was performed using Graphpad Prism 9. All statistical tests were two-tailed, and differences were considered statistically significant when *P* < 0.05. Normality tests were performed on behavior data using the Anderson–Darling test and Shapiro–Wilk test, and either parametric or non-parametric tests were applied accordingly. Non-parametric tests were used for coherence data.

## 3. Results

### 3.1. Novelty facilitates new learning

Our daily cognitive challenges often involve updating or replacing everyday routines with new information. We therefore tested behavioral performance in a task that required mice to modify an existing strategy. As previously reported (Park et al., 2021), we first trained mice to freely choose one of the two arms of a T-shaped maze to get a reward (free choice, Figure 1A). After 3 days of free choice training, mice developed a strong arm bias, efficiently choosing one arm consistently (Figure 1B; mice that will explore a familiar arena the next day: 92.7 ± 2.3%, mice that will explore a novel arena the next day: 89.1 ± 4.9%; both groups of mice made similar numbers of arm visits: 14.2 ± 1.2, 13.4 ± 0.7, respectively, Mann Whitney test, *P* = 0.9). The next day, the mice underwent flexible choice training in the same maze (Figure 1A). Here, they had to flexibly choose the goal not presented during the sample phase in a delayed-non-match-to-sample task (Figure 1C). The mice had to learn to overcome their arm bias established from free choice sessions for successful flexible choice performance (see section “2 Materials and methods” for details).

To explore the effect of novelty on learning, a group of mice explored a novel arena (Moncada and Viola, 2007; Ballarini et al., 2009) 1 h prior to the flexible choice task. As a control, a separate group of mice explored a familiar arena that the mice had experienced on 3 consecutive days prior to free choice sessions (Figure 1A). Novelty exposure itself did not elicit anxiety-related behavior (Park et al., 2021) (see section “2 Materials and methods”). Both groups of mice continued to choose their biased arm during the initial phase of flexible choice training. However, the mice exposed to novelty progressively overcame this bias, gradually performing better on trials when there was a mismatch between the goal arm and the biased arm (Figure 1D). This improvement was more rapid than that in mice exposed to the familiar environment (Figures 1D,E). To estimate at which trial mice learned the flexible choice rule, the inflection point of the learning curve was calculated. On average, the mice exposed to novelty acquired the new rule at trial 22 (Figure 1E). The average learning trial of the mice in the familiar group was not defined within 40 trials (Figure 1E). Together, these findings demonstrate that novel experience facilitates learning new rules, overcoming previously acquired old ones.

### 3.2. Novelty specifically impacts VTA-vHPC-mPFC circuitry

**FIGURE 2:**
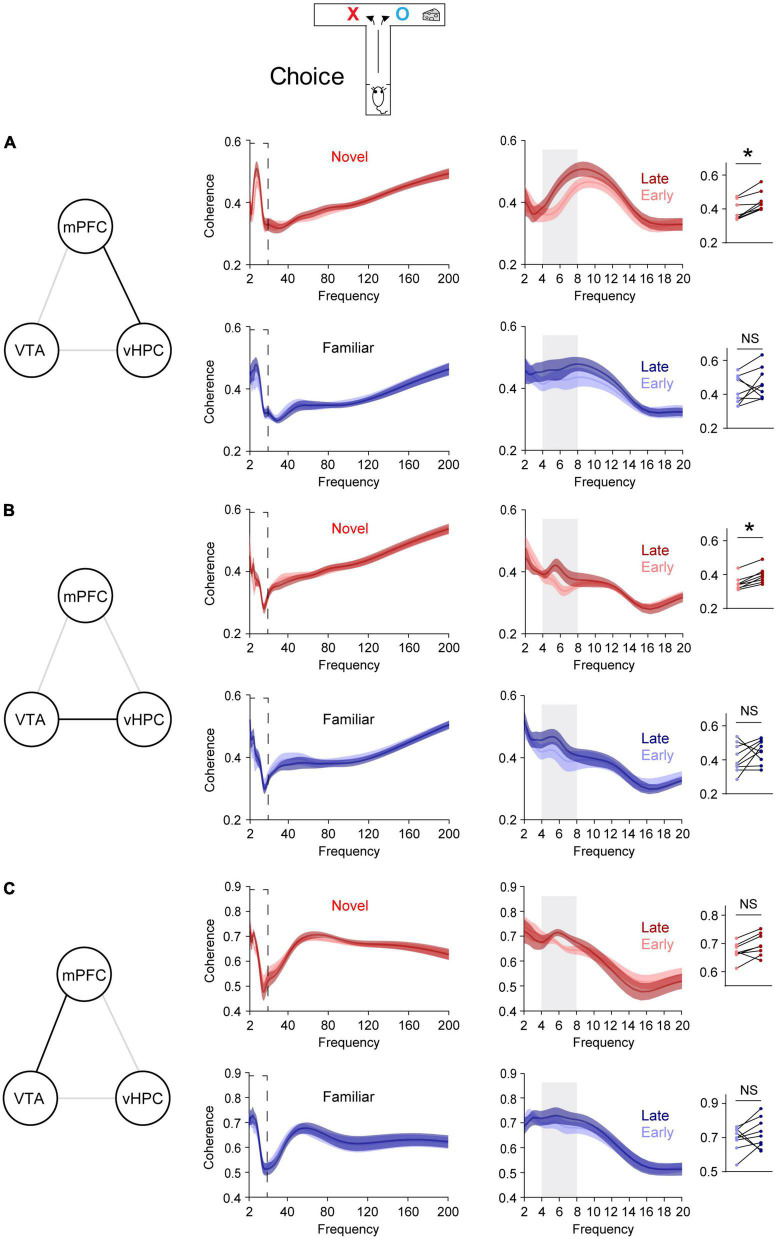
Novelty selectively permits learning-dependent theta coherence increase in the VTA-vHPC-mPFC circuit when mice flexibly choose a goal side. Coherence in the dashed box on the left is shown on the right. Dot plots show the average theta coherence of each mouse. **(A)** Mice exposed to the novel, but not the familiar, area displayed increased vHPC-mPFC theta coherence in late training compared with that of early training (Novel, *P* = 0.008; Familiar, *P* = 0.4). **(B)** The novel, but not the familiar, group showed increased VTA-vHPC theta coherence in late training compared with that of early training (Novel, *P* = 0.008; Familiar, *P* = 0.3). **(C)** Both novel and familiar groups exhibited similar mPFC-VTA theta coherence between the early and late choice phase of flexible choice training (H: Novel, *P* = 0.05; I: Familiar, *P* = 0.4). Wilcoxon signed-rank test. NS. not significant, novel (*n* = 8), familiar (*n* = 9), *P < 0.05. Data are represented as mean ± SEM.

A recent report demonstrates that novelty enhances new learning by permitting learning-dependent increases in synchrony between mPFC single neuronal activity and theta oscillations in the vHPC (Park et al., 2021). To further investigate this at a network level, synchrony was assessed using LFPs, which reflect collective local activity (Buzsáki et al., 2012), instead of single neuronal spiking. The hypothesis is that vHPC-mPFC theta coherence increases with learning. To test this, when mice were allowed to flexibly choose a goal (choice phase, Figure 1C), vHPC-mPFC coherence was compared before and after the average learning point of the novel group (Flexible choice trial 22, Figure 2A). Trials before and after trial 22 were defined as early and late phase, respectively (Figure 1E). In line with increased phase-locking of mPFC single neuronal activity to vHPC theta oscillations in late training (Park et al., 2021), the novel group displayed increased vHPC-mPFC theta coherence in late training, relative to that of early training (Figure 2A). On the other hand, this learning-dependent theta coherence increase was not observed in the familiar group (Figure 2A).

**FIGURE 3:**
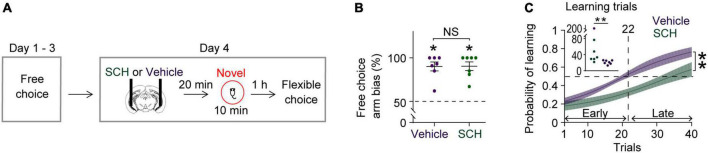
Blocking D1Rs in the vHPC prevents novelty-enhanced learning. **(A)** Experimental design. **(B)** Mice established an arm bias after 3 days of free choice sessions, before the administration of vehicle (*n* = 7) or SCH (*n* = 7) on day 4 (Wilcoxon signed-rank test, vehicle, *P* = 0.02; SCH, *P* = 0.02; vehicle vs. SCH, Mann Whitney test, *P* = 0.9). **(C)** Mice treated with SCH underperformed during flexible choice training compared with those treated with vehicle [Two-way RM ANOVA test, *F*_(1,12)_ = 13.25, *P* = 0.003]. The average learning trials were 22 (Vehicle) and 63 (SCH) (Mann–Whitney test, *P* = 0.004). Insets, learning trial of each mouse. *P < 0.05, **P < 0.005. Data are represented as mean ± SEM. **(C)** Adopted from Park et al. (2021).

Because dopaminergic inputs from the VTA convey novelty signal to the vHPC (Otmakhova et al., 2013) and the VTA densely projects to the vHPC (Park et al., 2021), VTA-vHPC coherence was measured (Figure 2B). The novel, but not the familiar, group showed increased VTA-vHPC theta coherence in late training compared with that of early training (Figure 2B). Moreover, regardless of the group, the degree of increases in theta coherence in late training in the vHPC-mPFC and VTA-vHPC circuit correlated with better learning (Supplementary Figure 3). Conversely, although the VTA projects to the mPFC (Morrens et al., 2020), both groups of mice did not show mPFC-VTA theta coherence changes through flexible choice training (Figure 2C).

To examine whether these findings are specific to learning, theta coherence was also measured when mice were guided to a goal, which does not involve choosing an arm based on learned information (sample phase, Figure 1C). In contrast to the above findings measured when mice freely chose a goal (choice phase, Figure 1C), neither group exhibited theta coherence changes through training in the VTA-vHPC-mPFC circuit in the sample phase (Supplementary Figure 4). Finally, theta coherence of circuits linked to the dHPC (dHPC-mPFC, VTA-dHPC, and vHPC-dHPC) was not affected by novelty, learning, or task phase (Supplementary Figures 5, 6). Overall, these findings suggest that novelty exposure permits learning-dependent connectivity strengthening specifically in the vHPC-mPFC and VTA-vHPC circuit.

### 3.3. D1-receptors in the vHPC mediate the effects of novelty

**FIGURE 4:**
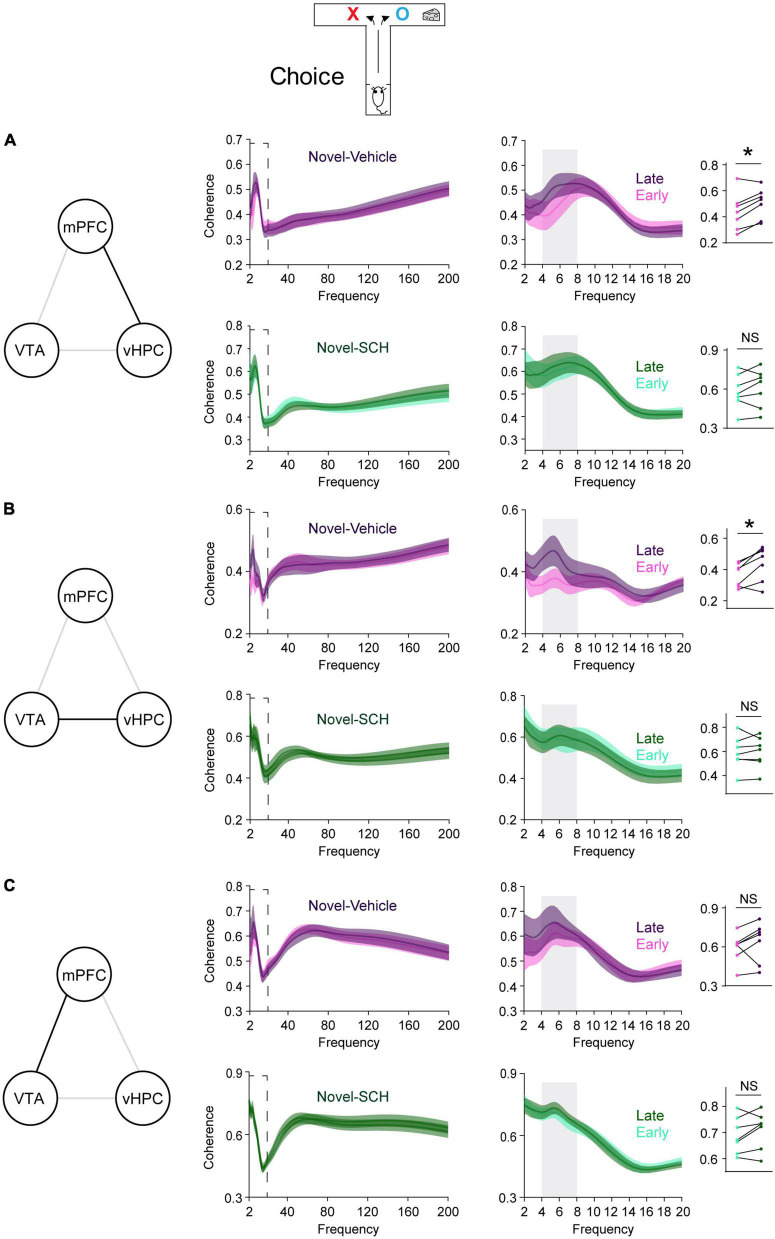
Blocking D1Rs in the vHPC prevents novelty-induced selective increases in theta coherence in the VTA-vHPC-mPFC circuit when mice flexibly choose a goal side. Coherence in the dashed box on the left is shown on the right. Dot plots show the average theta coherence of each mouse. **(A)** Mice treated with vehicle, but not SCH, displayed increased vHPC-mPFC theta coherence in late training compared with that of early training (vehicle, *P* = 0.03; SCH, *P* = 0.4). **(B)** The vehicle, but not the SCH, group showed increased VTA-vHPC theta coherence in late training compared with that of early training (vehicle, *P* = 0.03; SCH, *P* = 0.8). **(C)** Both vehicle and SCH groups exhibited similar mPFC-VTA theta coherence between the early and late choice phase of flexible choice training (vehicle, *P* = 0.3; SCH, *P* = 0.2). Vehicle (*n* = 7), SCH (*n* = 7). Wilcoxon signed-rank test. NS. not significant, *P < 0.05. Data are represented as mean ± SEM.

Blocking D1Rs in the vHPC has been shown to abolish novelty-enhanced learning (Park et al., 2021). Here, mice were infused with either the D1R-like antagonist SCH23390 (SCH) or vehicle specifically into the vHPC 20 min before novelty exposure (Figure 3A). These mice established arm bias after 3 days of free choice sessions (Figure 3B; vehicle: 90.1 ± 5.1%, SCH: 90.4 ± 5%; total number of arm visits: vehicle: 13.7 ± 0.6, SCH: 13.6 ± 0.6, Mann Whitney test, *P* = 0.9), and SCH treatment prevented mice from learning to overcome their arm bias compared with vehicle treatment (Figure 3C). These findings suggest that D1Rs in the vHPC may mediate the impact of novelty on learning-dependent connectivity strengthening in the vHPC-mPFC and VTA-vHPC circuit. To test this, circuit coherence was assessed when mice were allowed to flexibly choose a goal based on learned information (choice phase). SCH treatment blocked learning-dependent increases in theta coherence in the vHPC-mPFC and VTA-vHPC circuit compared with vehicle treatment (Figures 4A,B). The infusion of SCH or vehicle into the vHPC did not affect mPFC-VTA theta coherence through training (Figure 4C). Also, theta coherence of circuits linked to the dHPC (dHPC-mPFC, VTA-dHPC, and vHPC-dHPC) was not affected by drug treatment or learning (Supplementary Figure 7). Together, these findings confirm that D1Rs in the vHPC mediate the effects of novelty both behaviorally and physiologically.

Additionally, when assessing coherence in other frequency bands, only vHPC-mPFC and VTA-vHPC theta coherence showed consistent learning-dependent strengthening between the experiments in Figure 1 and those in Figure 3 (Supplementary Figures 8–10). Collectively, these findings demonstrate that novelty selectively permits plasticity in theta frequency in the vHPC-mPFC and VTA-vHPC circuit.

## 4. Discussion

Understanding the mechanism by which novel, salient experiences facilitate behavioral adaptation has implications for learning and memory in both health and disease. Previous work reports that novelty facilitates learning of task-relevant information by opening a plasticity window in the vHPC-mPFC circuit in theta frequency. The present study further demonstrates that novelty permits learning-associated strengthening of overall network connectivity in theta frequency in the vHPC-mPFC and VTA-vHPC, but not the mPFC-VTA circuit. Pharmacological blockage of D1Rs in the vHPC abolishes these specific effects of novelty in the VTA-vHPC-mPFC circuit. Notably, neither novelty, learning, or D1R blockage in the vHPC affect functional connectivity in theta frequency between the dHPC and other areas including the VTA, vHPC, and mPFC. Thus, we provide not only a new perspective on novelty-induced cognitive enhancements but also the circuit-level underpinnings of the selective effect of novelty.

The present study measured coherence in the VTA-vHPC-mPFC and VTA-dHPC-mPFC circuits to assess the impact of novelty on learning-dependent plasticity in these circuits. Previously, the impact of novelty on hippocampal-prefrontal circuitry was assessed using the spiking of single neurons (Park et al., 2021). This approach is optimal to achieve local specificity as it measures the spiking of individual neurons in the target area. As a complementary approach, the current study employed coherence, which reflects complex synaptic activity as well as synchronous neuronal spiking (Mysin and Shubina, 2022), to determine the network-level impact of novelty and learning on the brain circuit. Consistent with past findings using single neuronal activity (Park et al., 2021), novelty permits learning-dependent theta coherence increase in the vHPC-mPFC, but not the dHPC-mPFC circuit, further confirming that novelty selectively impacts the vHPC. Additionally, it is noteworthy that novelty allows learning-dependent increases in theta coherence in the VTA-vHPC, but not VTA-dHPC and mPFC-VTA circuit. This is striking because mPFC-VTA circuitry is implicated in learning enhancement following a novel odor presentation (Morrens et al., 2020) and the VTA projects to the dHPC and mPFC as well as the vHPC (Morrens et al., 2020; Park et al., 2021). In the vHPC, the VTA densely projects to areas CA1, CA3, and the dentate gyrus (Park et al., 2021). In the dHPC, on the other hand, the VTA densely projects to area CA3 and the dentate gyrus, and very sparsely to area CA1 (Park et al., 2021). Also, VTA neurons are highly heterogeneous in their function and projection targets (Aransay et al., 2015; Engelhard et al., 2019; Derdeyn et al., 2022). Therefore, it is plausible that different types of novelty exert a specific subset of VTA neurons that have distinct projection patterns across brain areas. Future experiments will confirm this speculation.

This study showed that D1Rs in the vHPC are required for novelty-enhanced learning and the associated physiology. The VTA is the primary source of dopamine in the vHPC, and novelty-induced coupling between the two structures predicts better memory (Adcock et al., 2006; Kempadoo et al., 2016). Moreover, VTA-vHPC interactions convey information about spatial novelty (Otmakhova et al., 2013). Thus, D1Rs in the vHPC are perfectly positioned to mediate learning-dependent connectivity strengthening in vHPC-mPFC and VTA-vHPC circuitry following novelty. Notably, we did not find a role for the dHPC in novelty-enhanced learning, even though inhibiting D1Rs in the dHPC impairs novelty-induced enhancement of memory retention (Lemon and Manahan-Vaughan, 2006; Takeuchi et al., 2016). In fact, dHPC CA1, and vHPC CA1 receive dense dopaminergic inputs from different sources; the latter from the VTA, and the former from the locus coeruleus, another area that is activated by novelty (Otmakhova et al., 2013; Kempadoo et al., 2016; Takeuchi et al., 2016). Thus, it is attractive to speculate that the dHPC and the vHPC differentially mediate novelty effects. The vHPC may underly the effects of novelty on learning, while the dHPC may do so for memory retention. However, we cannot rule out a secondary role for the dHPC in novelty-enhanced learning because dHPC place cells of mice lacking D1Rs display impaired spatial novelty detection (Tran et al., 2008). Future studies of hippocampal subregion-specific communication are required to parse out the relative contributions of hippocampal subregions to novelty-enhanced learning.

The anatomical projection patterns suggest that novelty activates dopamine neurons in the VTA, which in turn innervates D1Rs in the vHPC. Then the vHPC delivers this information to the mPFC *via* its unidirectional projection. Although the present data set favors this scenario, coherence measures have limitations because they represent overall connectivity strength and do not have directional information. Optogenetic manipulation of axon terminals will be necessary to directly confirm the direction of information flow triggered by novelty.

The VTA-vHPC-mPFC circuit is pivotal in learning and memory, and its pathology is found in a wide range of psychiatric disorders such as schizophrenia, depression, and autism (Colgin, 2011; Dichter et al., 2012; Li et al., 2015). Hence, this study provides new mechanistic insight for potential therapeutic interventions targeting the VTA-vHPC-mPFC circuit.

## Data availability statement

The original contributions presented in this study are included in the article/Supplementary material, further inquiries can be directed to the corresponding author.

## Ethics statement

The animal study was reviewed and approved by the Columbia University and the New York State Psychiatric Institute Institutional Animal Care and Use Committees (IACUC).

## Author contributions

AP conceived the study, designed and performed the experiments, analyzed the data, and wrote the manuscript.
