# Supplementary material for: Novelty selectively permits learning-associated plasticity in ventral tegmental-hippocampal-prefrontal circuitry
Source: Front Behav Neurosci. 2023 Jan 9;16:1091082. doi: 10.3389/fnbeh.2022.1091082 (PMC9868659; doi:10.3389/fnbeh.2022.1091082)
Supplement: Supplementary file 1 [file Data_Sheet_1.docx]

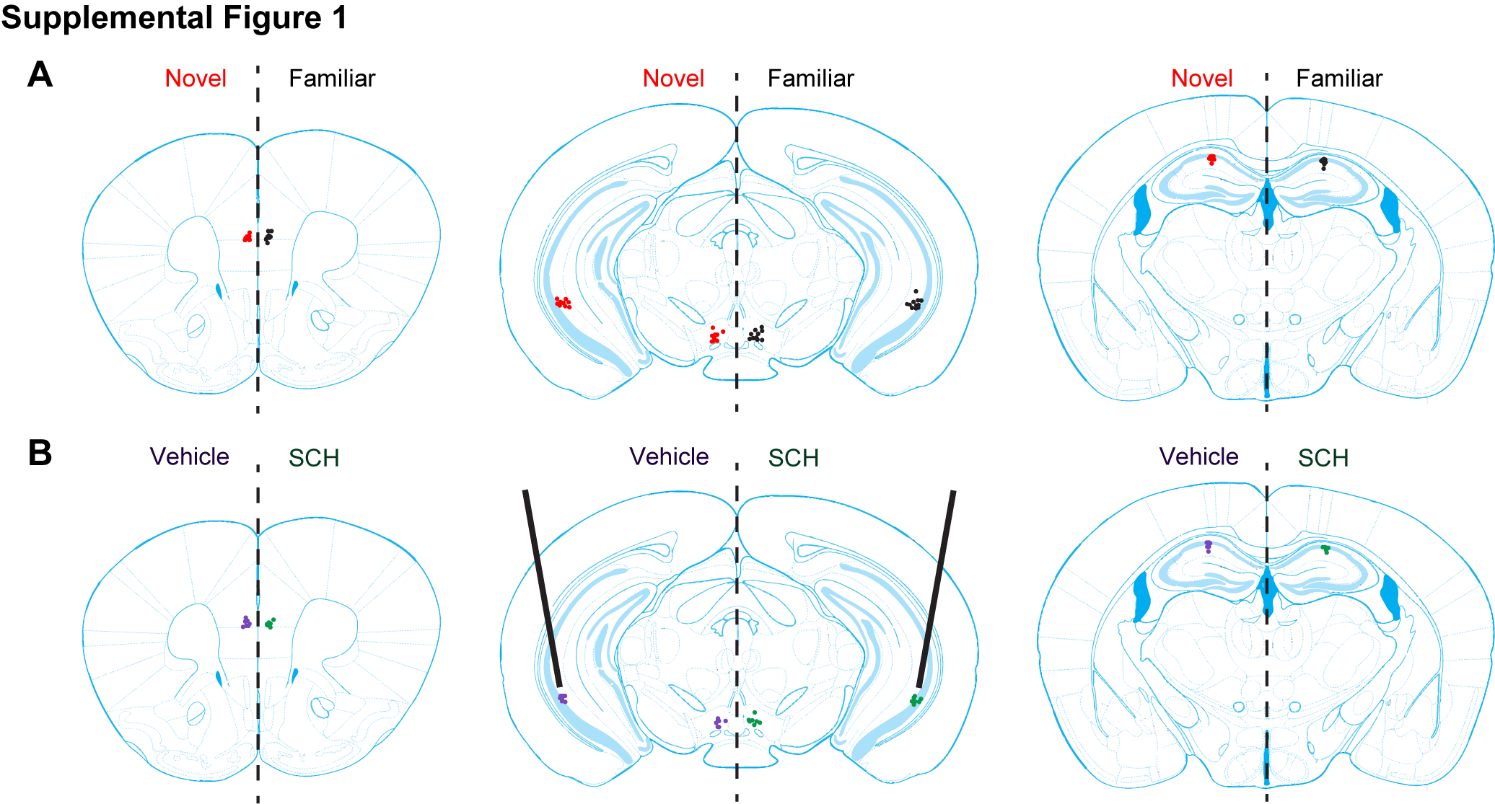
Supplementary Material

**
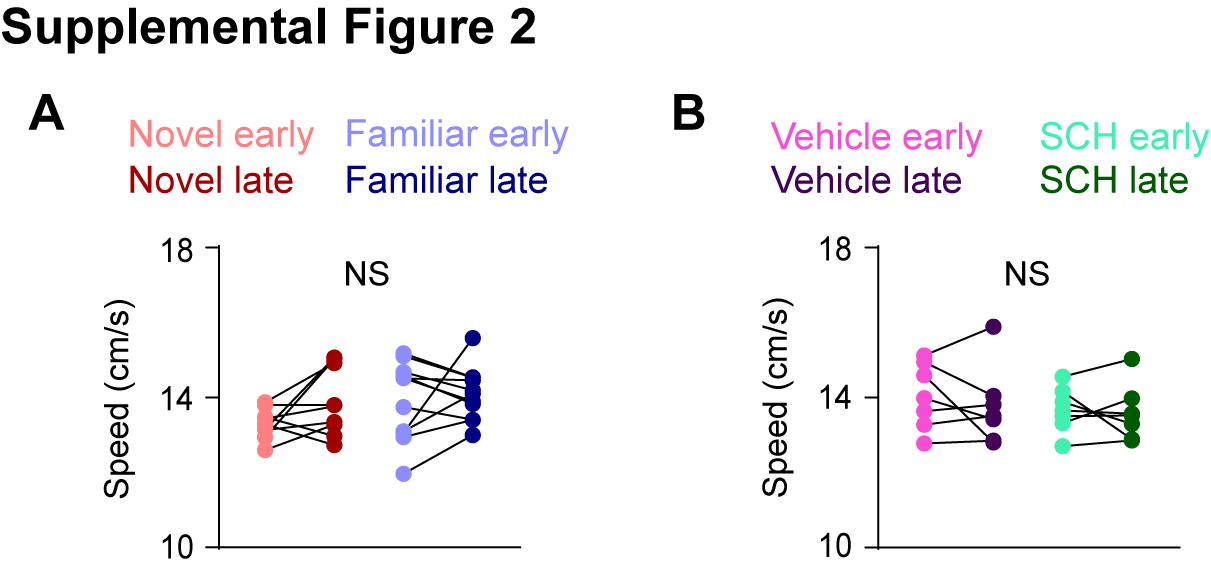
Supplementary Figure 1**. Electrode placements. (**A**) Novel vs. Familiar. (**B**) Novel-vehicle vs. Novel-SCH. Middle. Guide cannulae were inserted at 10°, and the tip of the field electrode was 0.7 mm below the tip of the guide cannulae.

**Supplementary Figure 2**. Running speed in the center arm during the choice phase. (**A**) Mice ran at comparable speed across all groups (One-way ANOVA test, F_(3,36)_ = 0.5, P = 0.2; Novel (n = 9), early (13.3 ± 0.1) vs. late (13.9 ± 0.3), t_(8)_ = 1.8, P = 0.1; Familiar (n = 11), early (13.9 ± 0.3) vs. late (14.1 ± 0.2), t_(10)_ = 0.2, P = 0.8). (**B**) Mice ran at comparable speed across all groups (One-way ANOVA test, F_(3,24)_ = 0.4, P = 0.8; Vehicle (n = 7), early (14.1 ± 0.3) vs. late (13.8 ± 0.4), t_(6)_ = 0.9, P = 0.4; SCH (n = 7), early (13.7 ± 0.2) vs. late (13.6 ± 0.3), t_(6)_ = 0.4, P = 0.7). NS. not significant. Group statistics are described as mean ± SEM.


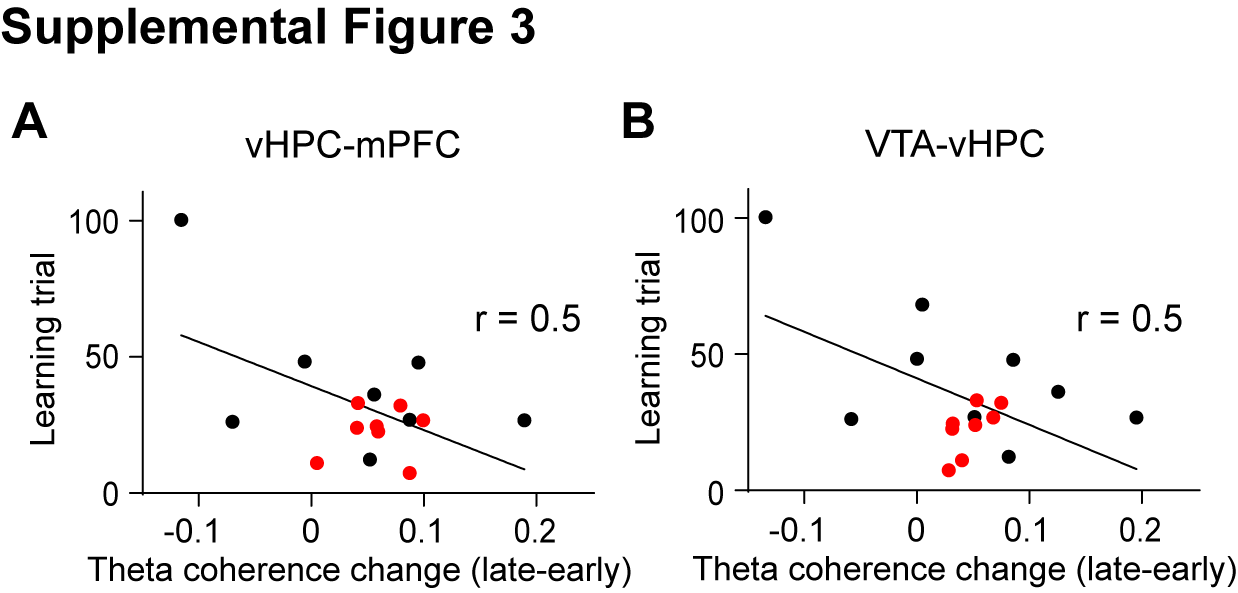


**Supplementary Figure 3**. Correlation between theta coherence changes during training and learning trial of each mouse. (**A**) Differences in vHPC-mPFC theta coherence between late and early training correlated with the trial number at which learning had occurred (Linear regression, F_(1,14)_ = 5.5, P = 0.03). (**B**) Differences in VTA-vHPC theta coherence between late and early training correlated with the trial number at which learning had occurred (Linear regression, F_(1,15)_ = 6, P = 0.03). Mice in the novel (red) and familiar (black) groups are combined.

**
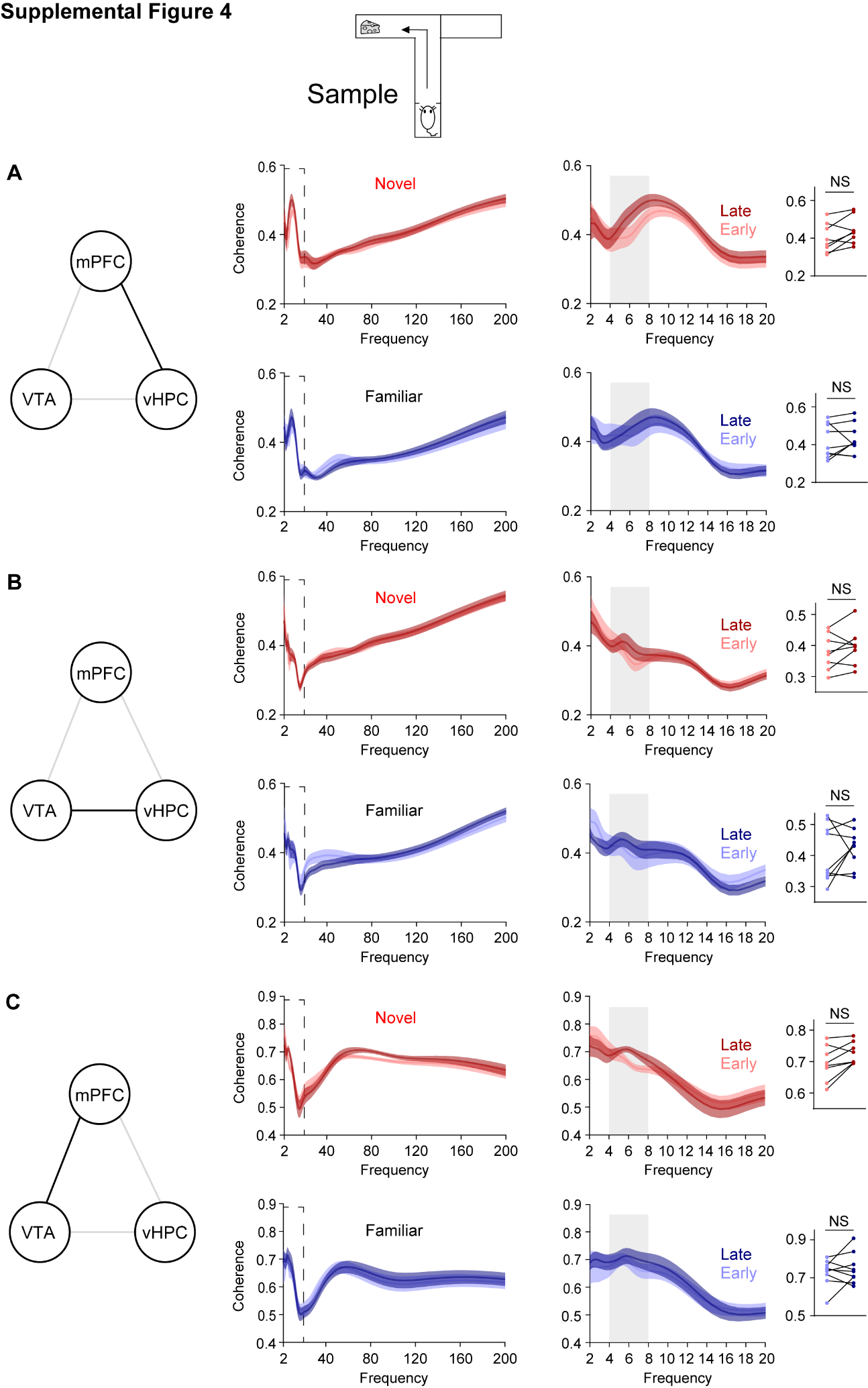
**

**Supplementary Figure 4**. Novelty does not affect theta coherence in the VTA-vHPC-mPFC circuit when mice are directed to a goal. Coherence in the dashed box on the left is shown on the right. Dot plots show the average theta coherence of each mouse. Mice explored either the novel or familiar arena displayed similar theta coherence between the early and late sample phase of flexible choice training in the vHPC-mPFC (**A**: Novel, P = 0.08; Familiar, P = 0.4), VTA-vHPC (**B**: Novel, P = 0.2; Familiar, P = 0.6), and mPFC-VTA circuit (**C**: Novel, P = 0.05; Familiar, P = 0.4). Wilcoxon signed-rank test. NS. not significant. Data are represented as mean ± SEM.


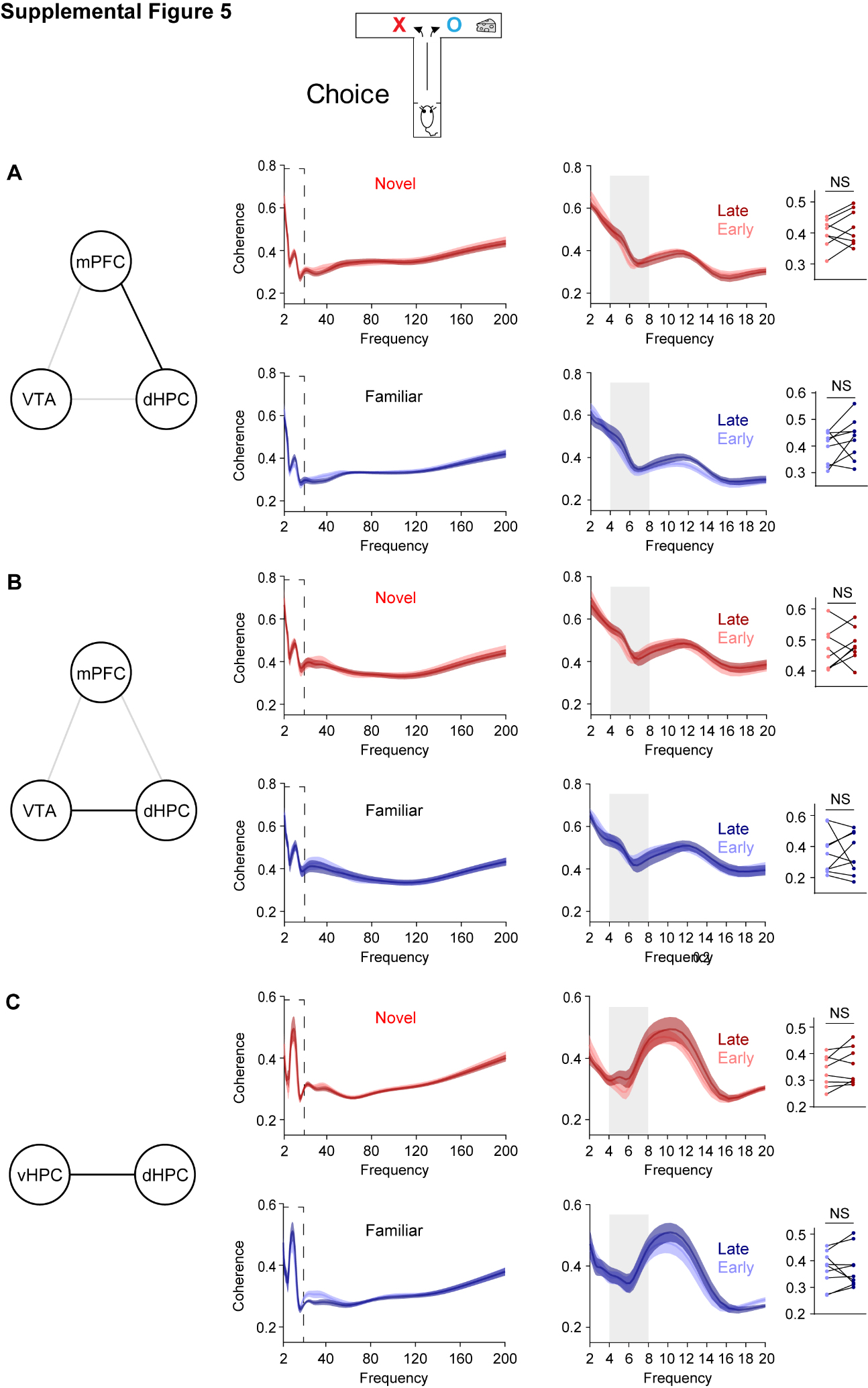


**Supplementary Figure 5**. Novelty does not affect coherence in the dHPC-mPFC, dHPC-VTA, and dHPC-vHPC circuit when mice flexibly choose a goal side. Coherence in the dashed box on the left is shown on the right. Dot plots show the average theta coherence of each mouse. Mice explored either a novel or familiar arena displayed similar theta coherence between the early and late choice phase of flexible choice training in the dHPC-mPFC (**A**: Novel, P = 0.1; Familiar, P = 0.2), VTA-dHPC (**B**: Novel, P = 0.6; Familiar, P = 0.8), and vHPC-dHPC circuit (**C**: Novel, P = 0.3; Familiar, P = 0.6). Wilcoxon signed-rank test. NS. not significant. Data are represented as mean ± SEM.

**
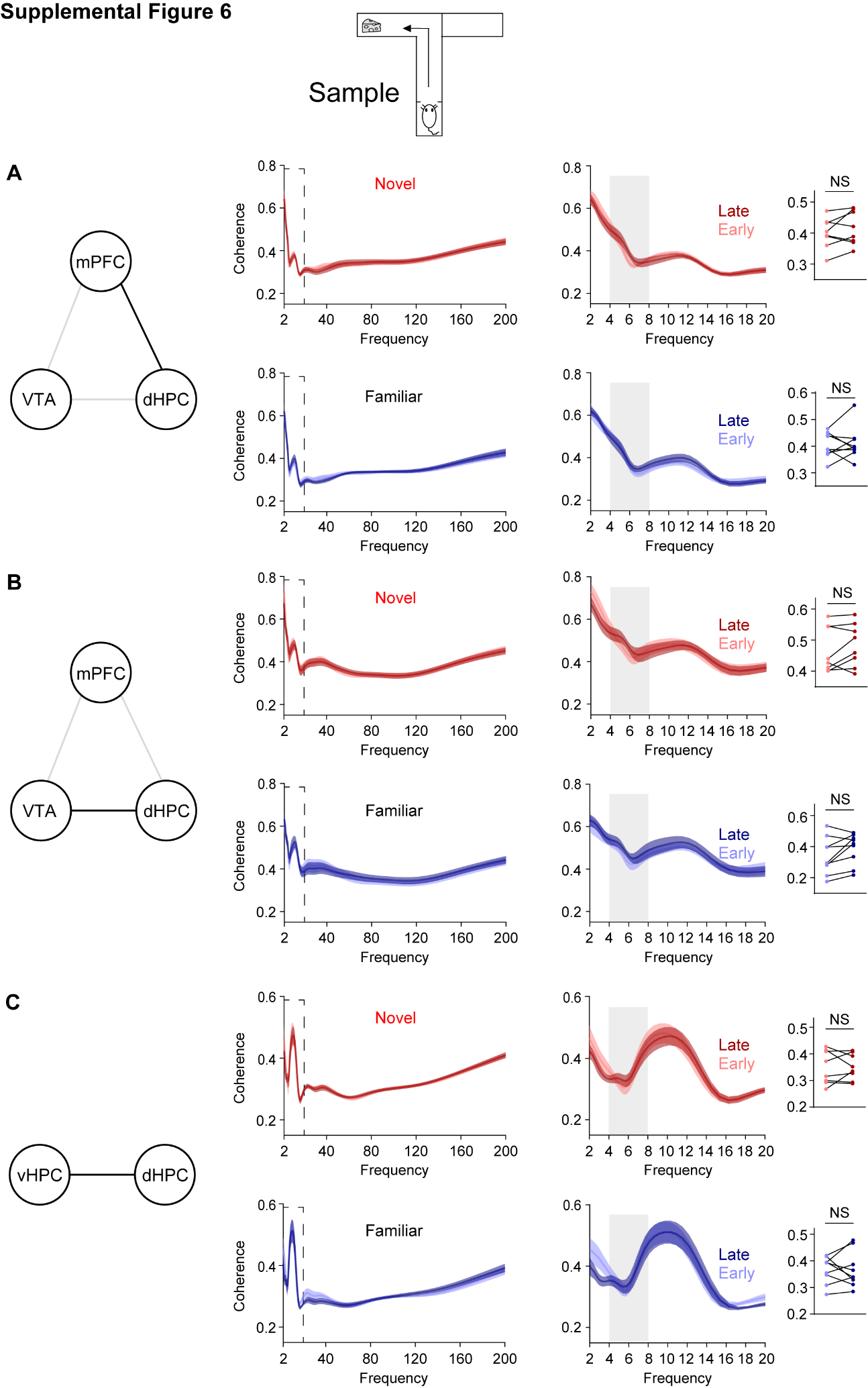
**

**Supplementary Figure 6**. Novelty does not affect coherence in the dHPC-mPFC, dHPC-VTA, and dHPC-vHPC circuit when mice are directed to a goal. Coherence in the dashed box on the left is shown on the right. Dot plots show the average theta coherence of each mouse. Mice explored either the novel or familiar arena displayed similar theta coherence between the early and late sample phase of flexible choice training in the dHPC-mPFC (**A**: Novel, P = 0.3; Familiar, P = 0.9), VTA-dHPC (**B**: Novel, P = 0.3; Familiar, P = 0.1), and vHPC-dHPC circuit (**C**: Novel, P = 0.9; Familiar, P = 0.9. Wilcoxon signed-rank test. NS. not significant. Data are represented as mean ± SEM.

**
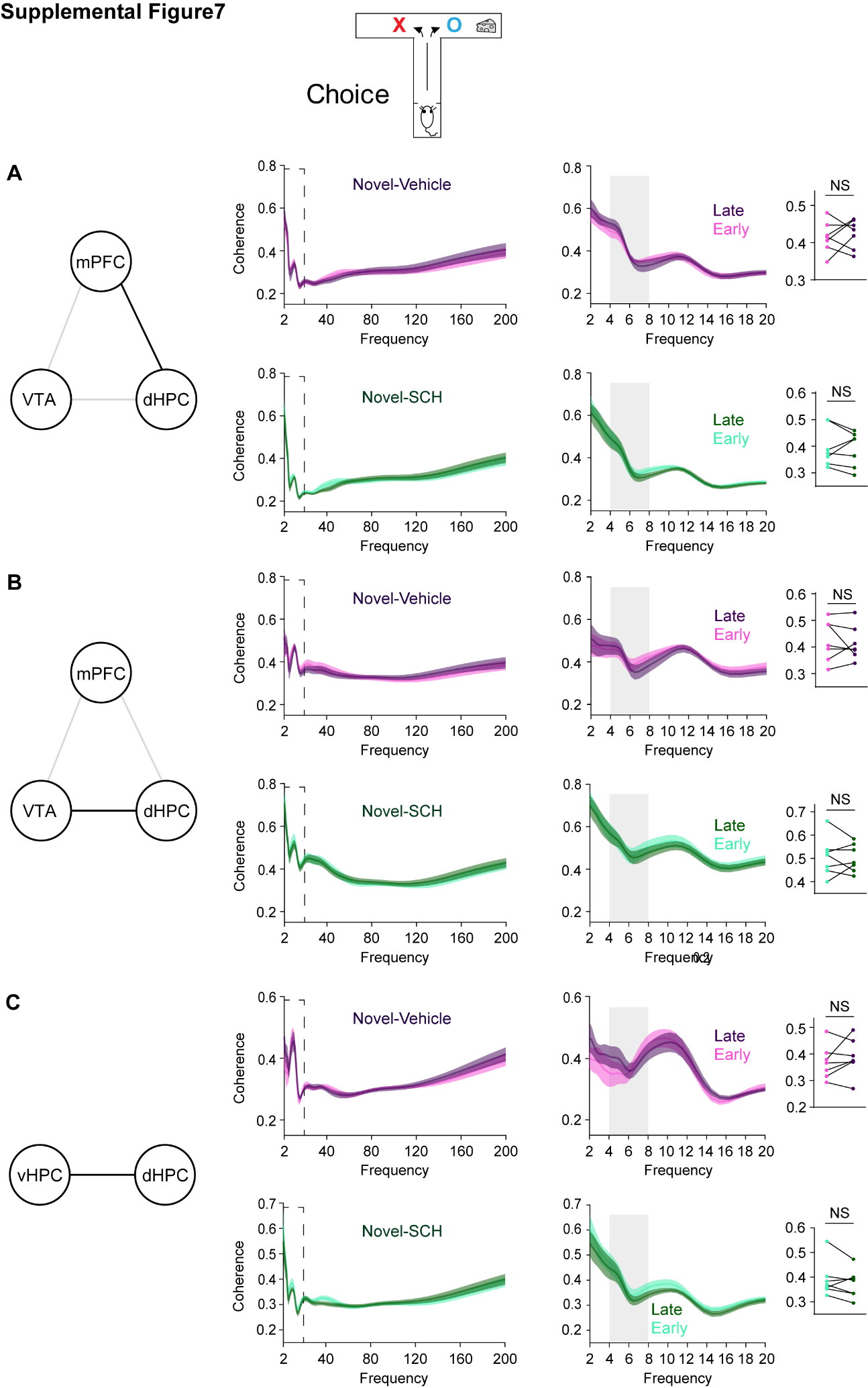
**

**Supplementary Figure 7**. Blocking D1Rs in the vHPC does not affect coherence in the dHPC-mPFC, dHPC-VTA, and dHPC-vHPC circuit when mice flexibly choose a goal side. Coherence in the dashed box on the left is shown on the right. Dot plots show the average theta coherence of each mouse. Mice treated with vehicle or SCH displayed similar theta coherence between the early and the late phase of flexible choice training in the dHPC-mPFC (**A**: Vehicle, P = 0.7; SCH, P = 0.8), VTA-dHPC (**B**: Vehicle, P = 0.9; SCH, P = 0.8), and vHPC-dHPC circuit (**C**: Vehicle, P = 0.5; SCH, P = 0.3). Wilcoxon signed-rank test. NS. not significant. Data are represented as mean ± SEM.

**
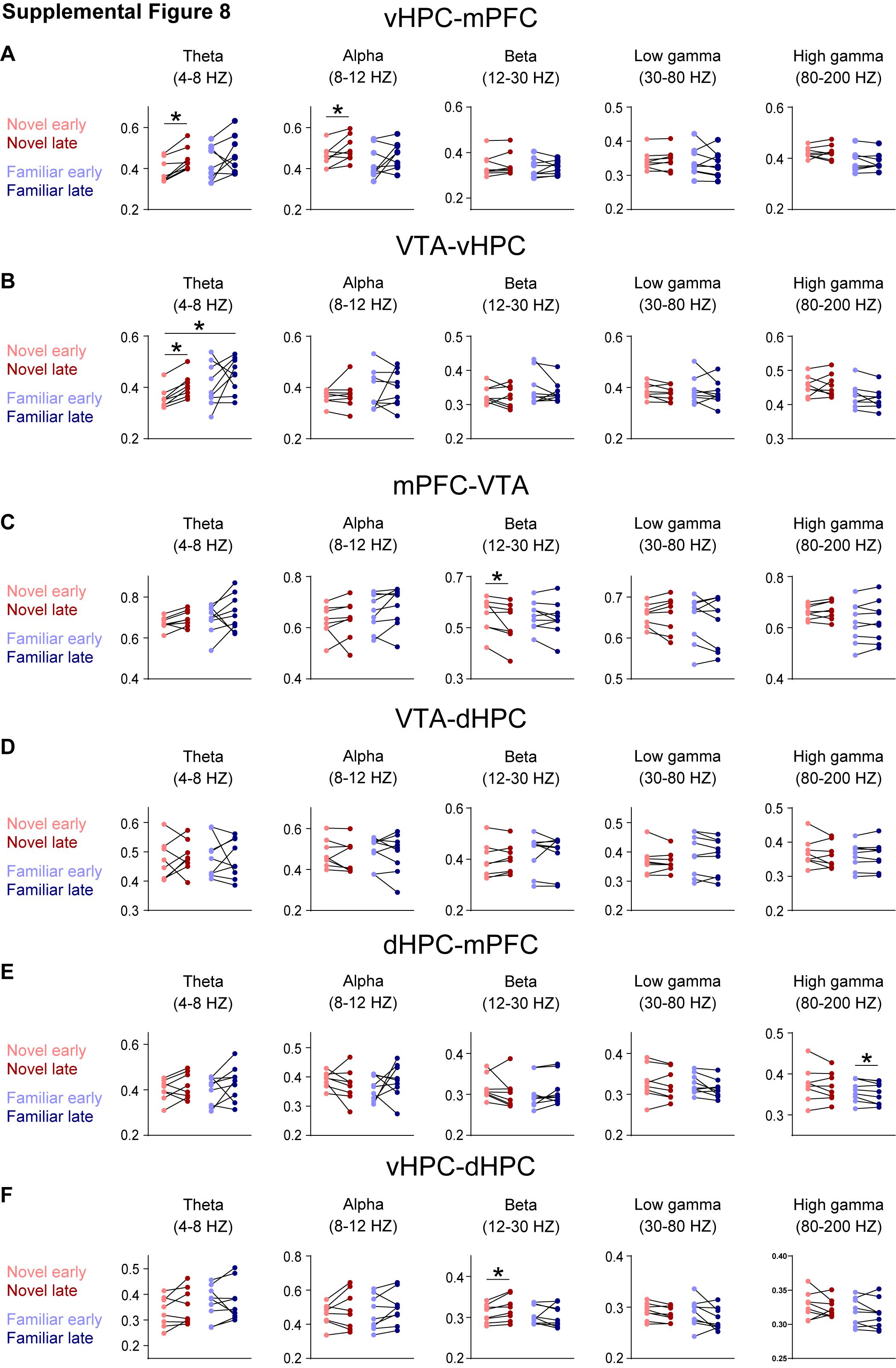
**

**Supplementary Figure 8**. Coherence changes across different frequency bands when mice flexibly choose a goal side (choice phase). (**A**) **vHPC-mPFC**. **Theta** (Novel: early (0.4 ± 0.02), late (0.4 ± 0.02); Familiar: early (0.4 ± 0.03), late (0.5 ± 0.03); Kruskal-Wallis test, P = 0.2; Novel early vs. late, P = 0.008; Familiar early vs. late, P = 0.4). **Alpha** (Novel: early (0.5 ± 0.02), late (0.5 ± 0.02); Familiar: early (0.4 ± 0.03), late (0.5 ± 0.02); Kruskal-Wallis test, P = 0.2; Novel early vs. late, P = 0.04; Familiar early vs. late, P = 0.3). **Beta** (Novel: early (0.3 ± 0.02), late (0.3 ± 0.02); Familiar: early (0.3 ± 0.01), late (0.3 ± 0.01); Kruskal-Wallis test, P = 0.8; Novel early vs. late, P = 0.1; Familiar early vs. late, P = 0.7). **Low gamma** (Novel: early (0.3 ± 0.01), late (0.3 ± 0.01); Familiar: early (0.3 ± 0.01), late (0.3 ± 0.01); Kruskal-Wallis test, P = 0.8; Novel early vs. late, P = 0.3; Familiar early vs. late, P = 0.4). **High gamma** (Novel: early (0.4 ± 0.007), late (0.4 ± 0.01); Familiar: early (0.4 ± 0.01), late (0.4 ± 0.01); Kruskal-Wallis test, P = 0.01; Novel early vs. late, P = 0.9; Familiar early vs. late, P = 0.9). (**B**) **VTA-vHPC**. **Theta** (Novel: early (0.4 ± 0.01), late (0.4 ± 0.02); Familiar: early (0.4 ± 0.03), late (0.4 ± 0.02); Kruskal-Wallis test, P = 0.03, Novel early vs. Familiar late, P = 0.02; Novel early vs. late, P = 0.008; Familiar early vs. late, P = 0.3). **Alpha** (Novel: early (0.4 ± 0.01), late (0.4 ± 0.02); Familiar: early (0.4 ± 0.03), late (0.4 ± 0.02); Kruskal-Wallis test, P = 0.9; Novel early vs. late, P = 0.7; Familiar early vs. late, P = 0.7). **Beta** (Novel: early (0.3 ± 0.01), late (0.3 ± 0.01); Familiar: early (0.3 ± 0.02), late (0.3 ± 0.01); Kruskal-Wallis test, P = 0.8; Novel early vs. late, P = 0.5; Familiar early vs. late, P = 0.5). **Low gamma** (Novel: early (0.4 ± 0.01), late (0.4 ± 0.01); Familiar: early (0.4 ± 0.02), late (0.4 ± 0.02); Kruskal-Wallis test, P = 0.9; Novel early vs. late, P = 0.2; Familiar early vs. late, P = 0.5). **High gamma** (Novel: early (0.5 ± 0.01), late (0.5 ± 0.01); Familiar: early (0.4 ± 0.01), late (0.4 ± 0.01); Kruskal-Wallis test, P = 0.02; Novel early vs. late, P = 0.9; Familiar early vs. late, P = 0.4). (**C**) **mPFC-VTA**. **Theta** (Novel: early (0.7 ± 0.01), late (0.7 ± 0.01); Familiar: early (0.7 ± 0.02), late (0.7 ± 0.03); Kruskal-Wallis test, P = 0.5; Novel early vs. late, P = 0.05; Familiar early vs. late, P = 0.4). **Alpha** (Novel: early (0.6 ± 0.02), late (0.6 ± 0.03); Familiar: early (0.7 ± 0.02), late (0.7 ± 0.03); Kruskal-Wallis test, P = 0.2; Novel early vs. late, P = 0.2; Familiar early vs. late, P = 0.1). **Beta** (Novel: early (0.5 ± 0.02), late (0.5 ± 0.03); Familiar: early (0.5 ± 0.02), late (0.5 ± 0.02); Kruskal-Wallis test, P = 0.8; Novel early vs. late, P = 0.02; Familiar early vs. late, P = 0.4). **Low gamma** (Novel: early (0.7 ± 0.01), late (0.7 ± 0.02); Familiar: early (0.6 ± 0.02), late (0.6 ± 0.02); Kruskal-Wallis test, P = 0.9; Novel early vs. late, P = 0.7; Familiar early vs. late, P = 0.5). **High gamma** (Novel: early (0.7 ± 0.01), late (0.7 ± 0.01); Familiar: early (0.6 ± 0.03), late (0.6 ± 0.03); Kruskal-Wallis test, P = 0.3; Novel early vs. late, P = 0.3; Familiar early vs. late, P = 0.4). (**D**) **VTA-dHPC**. **Theta** (Novel: early (0.5 ± 0.02), late (0.5 ± 0.02); Familiar: early (0.5 ± 0.02), late (0.5 ± 0.02); Kruskal-Wallis test, P = 0.9; Novel early vs. late, P = 0.6; Familiar early vs. late, P = 0.8). **Alpha** (Novel: early (0.5 ± 0.02), late (0.5 ± 0.03); Familiar: early (0.5 ± 0.02), late (0.5 ± 0.03); Kruskal-Wallis test, P = 0.9; Novel early vs. late, P = 0.3; Familiar early vs. late, P = 0.6). **Beta** (Novel: early (0.4 ± 0.02), late (0.4 ± 0.02); Familiar: early (0.4 ± 0.02), late (0.4 ± 0.02); Kruskal-Wallis test, P = 0.5; Novel early vs. late, P = 0.3; Familiar early vs. late, P = 0.7). **Low gamma** (Novel: early (0.4 ± 0.02), late (0.4 ± 0.01); Familiar: early (0.4 ± 0.02), late (0.4 ± 0.02); Kruskal-Wallis test, P = 0.8; Novel early vs. late, P = 0.5; Familiar early vs. late, P = 0.3). **High gamma** (Novel: early (0.4 ± 0.01), late (0.4 ± 0.01); Familiar: early (0.4 ± 0.01), late (0.4 ± 0.01); Kruskal-Wallis test, P = 0.9; Novel early vs. late, P = 0.4; Familiar early vs. late, P = 0.9). (**E**) **dHPC-mPFC**. **Theta** (Novel: early (0.4 ± 0.02), late (0.4 ± 0.02); Familiar: early (0.4 ± 0.02), late (0.4 ± 0.03); Kruskal-Wallis test, P = 0.7; Novel early vs. late, P = 0.1; Familiar early vs. late, P = 0.2). **Alpha** (Novel: early (0.4 ± 0.01), late (0.4 ± 0.02); Familiar: early (0.4 ± 0.01), late (0.4 ± 0.02); Kruskal-Wallis test, P = 0.5; Novel early vs. late, P = 0.2; Familiar early vs. late, P = 0.5). **Beta** (Novel: early (0.3 ± 0.01), late (0.3 ± 0.01); Familiar: early (0.3 ± 0.01), late (0.3 ± 0.01); Kruskal-Wallis test, P = 0.5; Novel early vs. late, P = 0.2; Familiar early vs. late, P = 0.09). **Low gamma** (Novel: early (0.3 ± 0.01), late (0.3 ± 0.01); Familiar: early (0.3 ± 0.01), late (0.3 ± 0.01); Kruskal-Wallis test, P = 0.7; Novel early vs. late, P = 0.5; Familiar early vs. late, P = 0.05). **High gamma** (Novel: early (0.4 ± 0.02), late (0.4 ± 0.01); Familiar: early (0.4 ± 0.01), late (0.4 ± 0.01); Kruskal-Wallis test, P = 0.7; Novel early vs. late, P = 0.5; Familiar early vs. late, P = 0.02). (**F**) **vHPC-dHPC**. **Theta** (Novel: early (0.3 ± 0.02), late (0.4 ± 0.02); Familiar: early (0.4 ± 0.02), late (0.4 ± 0.02); Kruskal-Wallis test, P = 0.7; Novel early vs. late, P = 0.3; Familiar early vs. late, P = 0.6). **Alpha** (Novel: early (0.5 ± 0.02), late (0.5 ± 0.04); Familiar: early (0.5 ± 0.03), late (0.5 ± 0.03); Kruskal-Wallis test, P = 0.9; Novel early vs. late, P = 0.5; Familiar early vs. late, P = 0.1). **Beta** (Novel: early (0.3 ± 0.01), late (0.3 ± 0.01); Familiar: early (0.3 ± 0.01), late (0.3 ± 0.01); Kruskal-Wallis test, P = 0.6; Novel early vs. late, P = 0.01; Familiar early vs. late, P = 0.3). **Low gamma** (Novel: early (0.3 ± 0.006), late (0.3 ± 0.005); Familiar: early (0.3 ± 0.009), late (0.3 ± 0.007); Kruskal-Wallis test, P = 0.3; Novel early vs. late, P = 0.05; Familiar early vs. late, P = 0.2). **High gamma** (Novel: early (0.3 ± 0.007), late (0.3 ± 0.005); Familiar: early (0.3 ± 0.007), late (0.3 ± 0.007); Kruskal-Wallis test, P = 0.5; Novel early vs. late, P = 0.5; Familiar early vs. late, P = 0.4). Wilcoxon signed-rank tests were used for paired analyses within the novel or familiar group. Kruskal-Wallis test was used for between group analyses. Dunn’s post hoc test was used where applicable. Novel (n = 8), Familiar (n = 9). * P < 0.05. Group statistics are described as mean ± SEM.

**
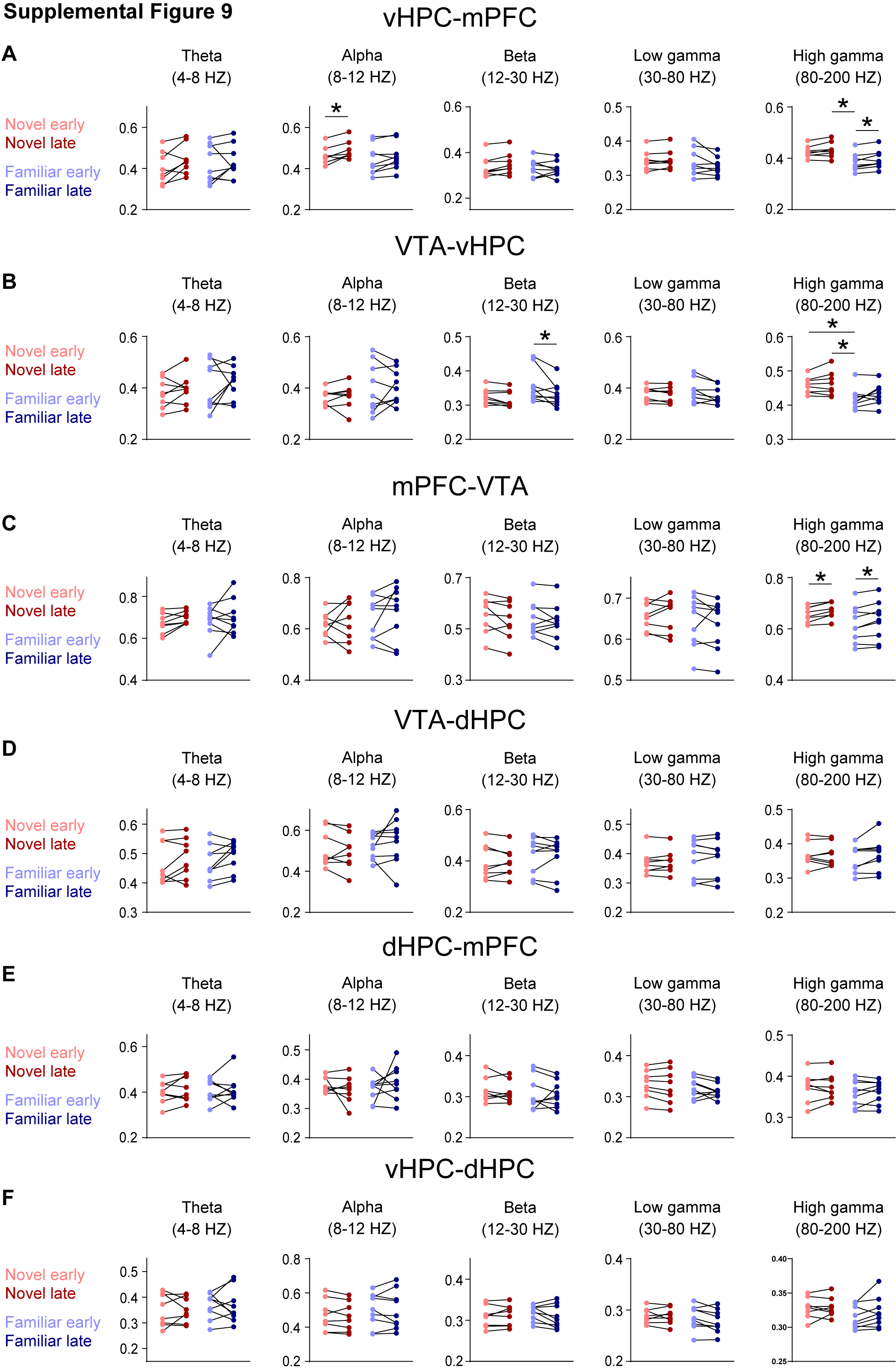
**

**Supplementary Figure 9**. Coherence changes across different frequency bands when mice are directed to a goal (sample phase). (**A**) **vHPC-mPFC**. **Theta** (Novel: early (0.4 ± 0.03), late (0.4 ± 0.03); Familiar: early (0.4 ± 0.03), late (0.4 ± 0.03); Kruskal-Wallis test, P = 0.6; Novel early vs. late, P = 0.08; Familiar early vs. late, P = 0.4). **Alpha** (Novel: early (0.5 ± 0.02), late (0.5 ± 0.02); Familiar: early (0.4 ± 0.02), late (0.5 ± 0.02); Kruskal-Wallis test, P = 0.3; Novel early vs. late, P = 0.02; Familiar early vs. late, P = 0.07). **Beta** (Novel: early (0.3 ± 0.02), late (0.4 ± 0.02); Familiar: early (0.3 ± 0.01), late (0.3 ± 0.01); Kruskal-Wallis test, P = 0.7; Novel early vs. late, P = 0.5; Familiar early vs. late, P = 0.9). **Low gamma** (Novel: early (0.3 ± 0.01), late (0.3 ± 0.01); Familiar: early (0.3 ± 0.01), late (0.3 ± 0.01); Kruskal-Wallis test, P = 0.3; Novel early vs. late, P = 0.4; Familiar early vs. late, P = 0.2). **High gamma** (Novel: early (0.4 ± 0.008), late (0.4 ± 0.01); Familiar: early (0.4 ± 0.01), late (0.4 ± 0.01); Kruskal-Wallis test, P = 0.006, Novel late vs. Familiar early, P = 0.02; Novel early vs. late, P = 0.1; Familiar early vs. late, P = 0.03). (**B**) **VTA-vHPC**. **Theta** (Novel: early (0.4 ± 0.02), late (0.4 ± 0.02); Familiar: early (0.4 ± 0.03), late (0.4 ± 0.02); Kruskal-Wallis test, P = 0.6; Novel early vs. late, P = 0.2; Familiar early vs. late, P = 0.6). **Alpha** (Novel: early (0.4 ± 0.01), late (0.4 ± 0.02); Familiar: early (0.4 ± 0.03), late (0.4 ± 0.02); Kruskal-Wallis test, P = 0.8; Novel early vs. late, P = 0.4; Familiar early vs. late, P = 0.9). **Beta** (Novel: early (0.3 ± 0.008), late (0.3 ± 0.008); Familiar: early (0.4 ± 0.02), late (0.3 ± 0.01); Kruskal-Wallis test, P = 0.8; Novel early vs. late, P = 0.5; Familiar early vs. late, P = 0.5). **Low gamma** (Novel: early (0.4 ± 0.01), late (0.4 ± 0.01); Familiar: early (0.4 ± 0.01), late (0.4 ± 0.01); Kruskal-Wallis test, P = 0.8; Novel early vs. late, P = 0.1; Familiar early vs. late, P = 0.05). **High gamma** (Novel: early (0.5 ± 0.008), late (0.5 ± 0.01); Familiar: early (0.4 ± 0.01), late (0.4 ± 0.01); Kruskal-Wallis test, P = 0.01, Novel early vs. Familiar early, P = 0.04, Novel late vs. Familiar early, P = 0.04; Novel early vs. late, P = 0.5; Familiar early vs. late, P = 0.3). (**C**) **mPFC-VTA**. **Theta** (Novel: early (0.7 ± 0.02), late (0.7 ± 0.01); Familiar: early (0.7 ± 0.02), late (0.7 ± 0.03); Kruskal-Wallis test, P = 0.6; Novel early vs. late, P = 0.05; Familiar early vs. late, P = 0.9). **Alpha** (Novel: early (0.6 ± 0.02), late (0.6 ± 0.03); Familiar: early (0.6 ± 0.03), late (0.7 ± 0.03); Kruskal-Wallis test, P = 0.7; Novel early vs. late, P = 0.9; Familiar early vs. late, P = 0.7). **Beta** (Novel: early (0.6 ± 0.03), late (0.5 ± 0.03); Familiar: early (0.5 ± 0.02), late (0.5 ± 0.02); Kruskal-Wallis test, P = 0.9; Novel early vs. late, P = 0.1; Familiar early vs. late, P = 0.4). **Low gamma** (Novel: early (0.7 ± 0.01), late (0.7 ± 0.02); Familiar: early (0.6 ± 0.02), late (0.6 ± 0.02); Kruskal-Wallis test, P = 0.8; Novel early vs. late, P = 0.7; Familiar early vs. late, P = 0.2). **High gamma** (Novel: early (0.7 ± 0.01), late (0.7 ± 0.01); Familiar: early (0.6 ± 0.02), late (0.6 ± 0.02); Kruskal-Wallis test, P = 0.2; Novel early vs. late, P = 0.08; Familiar early vs. late, P = 0.04). (**D**) **VTA-dHPC**. **Theta** (Novel: early (0.5 ± 0.03), late (0.5 ± 0.02); Familiar: early (0.5 ± 0.02), late (0.5 ± 0.02); Kruskal-Wallis test, P = 0.8; Novel early vs. late, P = 0.3; Familiar early vs. late, P = 0.1). **Alpha** (Novel: early (0.5 ± 0.03), late (0.5 ± 0.03); Familiar: early (0.5 ± 0.02), late (0.5 ± 0.03); Kruskal-Wallis test, P = 0.6; Novel early vs. late, P = 0.5; Familiar early vs. late, P = 0.2). **Beta** (Novel: early (0.4 ± 0.02), late (0.4 ± 0.02); Familiar: early (0.4 ± 0.02), late (0.4 ± 0.02); Kruskal-Wallis test, P = 0.7; Novel early vs. late, P = 0.9; Familiar early vs. late, P = 0.6). **Low gamma** (Novel: early (0.4 ± 0.01), late (0.4 ± 0.01); Familiar: early (0.4 ± 0.02), late (0.4 ± 0.02); Kruskal-Wallis test, P = 0.9; Novel early vs. late, P = 0.6; Familiar early vs. late, P = 0.9). **High gamma** (Novel: early (0.4 ± 0.01), late (0.4 ± 0.01); Familiar: early (0.4 ± 0.01), late (0.4 ± 0.01); Kruskal-Wallis test, P = 0.9; Novel early vs. late, P = 0.9; Familiar early vs. late, P = 0.05). (**E**) **dHPC-mPFC**. **Theta** (Novel: early (0.4 ± 0.02), late (0.4 ± 0.02); Familiar: early (0.4 ± 0.02), late (0.4 ± 0.02); Kruskal-Wallis test, P = 0.9; Novel early vs. late, P = 0.3; Familiar early vs. late, P = 0.9). **Alpha** (Novel: early (0.4 ± 0.01), late (0.4 ± 0.02); Familiar: early (0.4 ± 0.01), late (0.4 ± 0.02); Kruskal-Wallis test, P = 0.8; Novel early vs. late, P = 0.7; Familiar early vs. late, P = 0.5). **Beta** (Novel: early (0.3 ± 0.01), late (0.3 ± 0.01); Familiar: early (0.3 ± 0.01), late (0.3 ± 0.01); Kruskal-Wallis test, P = 0.7; Novel early vs. late, P = 0.8; Familiar early vs. late, P = 0.5). **Low gamma** (Novel: early (0.3 ± 0.01), late (0.3 ± 0.01); Familiar: early (0.3 ± 0.01), late (0.3 ± 0.006); Kruskal-Wallis test, P = 0.6; Novel early vs. late, P = 0.4; Familiar early vs. late, P = 0.3). **High gamma** (Novel: early (0.4 ± 0.01), late (0.4 ± 0.01); Familiar: early (0.4 ± 0.01), late (0.4 ± 0.009); Kruskal-Wallis test, P = 0.8; Novel early vs. late, P = 0.8; Familiar early vs. late, P = 0.9). (**F**) **vHPC-dHPC**. **Theta** (Novel: early (0.4 ± 0.02), late (0.4 ± 0.02); Familiar: early (0.4 ± 0.02), late (0.4 ± 0.02); Kruskal-Wallis test, P = 0.9; Novel early vs. late, P = 0.9; Familiar early vs. late, P = 0.9). **Alpha** (Novel: early (0.5 ± 0.03), late (0.5 ± 0.03); Familiar: early (0.5 ± 0.03), late (0.5 ± 0.04); Kruskal-Wallis test, P = 0.8; Novel early vs. late, P = 0.4; Familiar early vs. late, P = 0.9). **Beta** (Novel: early (0.3 ± 0.009), late (0.3 ± 0.009); Familiar: early (0.3 ± 0.006), late (0.3 ± 0.009); Kruskal-Wallis test, P = 0.9; Novel early vs. late, P = 0.5; Familiar early vs. late, P = 0.3). **Low gamma** (Novel: early (0.3 ± 0.005), late (0.3 ± 0.006); Familiar: early (0.3 ± 0.008), late (0.3 ± 0.007); Kruskal-Wallis test, P = 0.7; Novel early vs. late, P = 0.9; Familiar early vs. late, P = 0.07). **High gamma** (Novel: early (0.3 ± 0.005), late (0.3 ± 0.005); Familiar: early (0.3 ± 0.005), late (0.3 ± 0.007); Kruskal-Wallis test, P = 0.2; Novel early vs. late, P = 0.9; Familiar early vs. late, P = 0.07). Wilcoxon signed-rank tests were used for paired analyses within the novel or familiar group. Kruskal-Wallis test was used for between group analyses. Dunn’s post hoc test was used where applicable. Novel (n = 8), Familiar (n = 9). * P < 0.05. Group statistics are described as mean ± SEM.

**
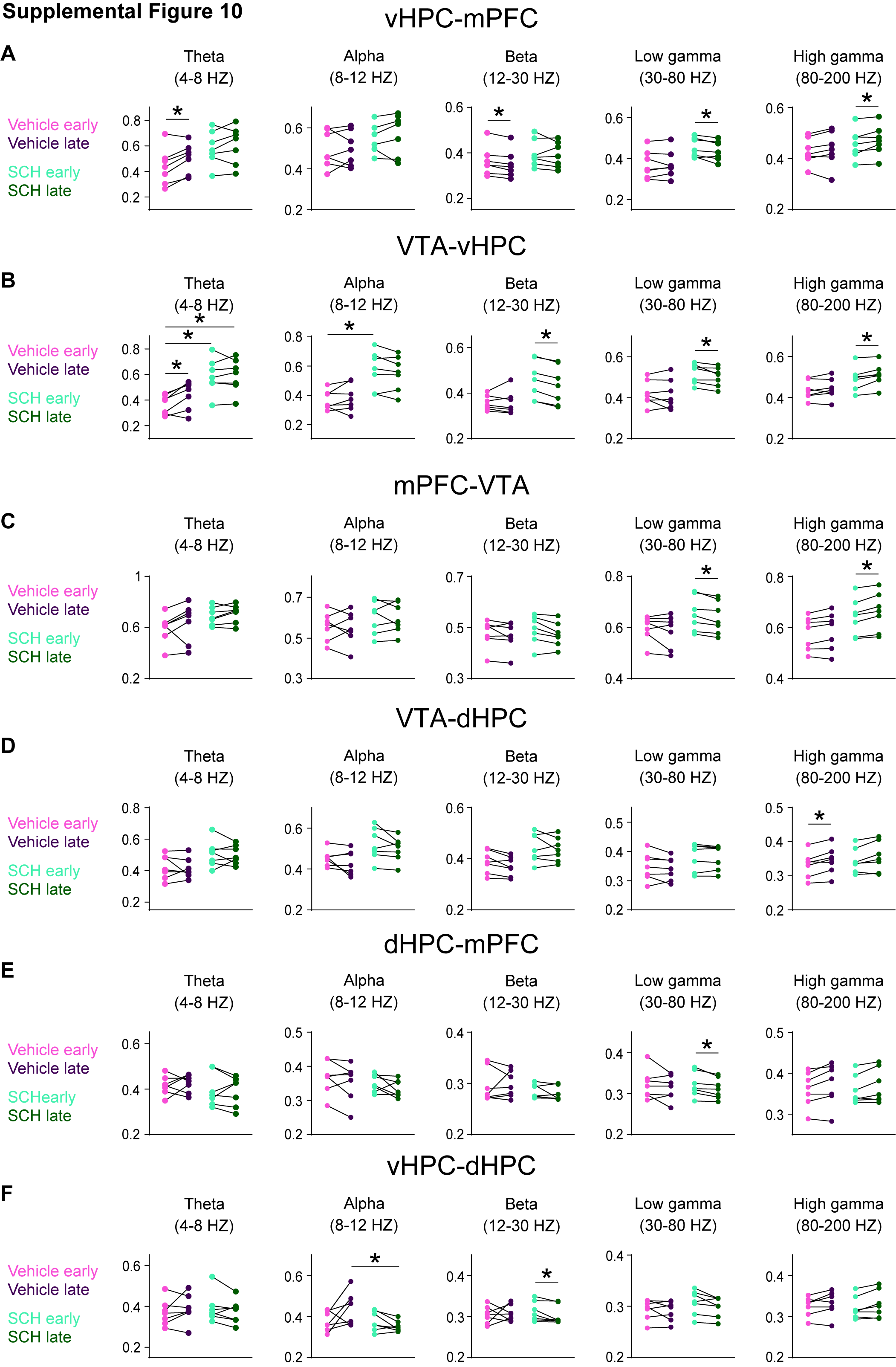
**

**Supplementary Figure 10**. Coherence changes across different frequency bands when mice treated with vehicle or SCH flexibly choose a goal side (choice phase). (**A**) **vHPC-mPFC**. **Theta** (Vehicle: early (0.4 ± 0.05), late (0.5 ± 0.04); SCH: early (0.6 ± 0.06), late (0.6 ± 0.06); Kruskal-Wallis test, P = 0.1; Vehicle early vs. late, P = 0.03; SCH early vs. late, P = 0.4). **Alpha** (Vehicle: early (0.5 ± 0.02), late (0.5 ± 0.02); SCH: early (0.6 ± 0.02), late (0.6 ± 0.02); Kruskal-Wallis test, P = 0.2; Vehicle early vs. late, P = 0.9; SCH early vs. late, P = 0.9). **Beta** (Vehicle: early (0.4 ± 0.02), late (0.4 ± 0.02); SCH: early (0.4 ± 0.01), late (0.4 ± 0.01); Kruskal-Wallis test, P = 0.3; Vehicle early vs. late, P = 0.02; SCH early vs. late, P = 0.5). **Low gamma** (Vehicle: early (0.4 ± 0.03), late (0.4 ± 0.03); SCH: early (0.5 ± 0.02), late (0.4 ± 0.02); Kruskal-Wallis test, P = 0.03; Vehicle early vs. late, P = 0.8; SCH early vs. late, P = 0.03). **High gamma** (Vehicle: early (0.4 ± 0.02), late (0.4 ± 0.03); SCH: early (0.5 ± 0.02), late (0.5 ± 0.02); Kruskal-Wallis test, P = 0.6; Vehicle early vs. late, P = 0.4; SCH early vs. late, P = 0.03). (**B**) **VTA-vHPC**. **Theta** (Vehicle: early (0.4 ± 0.03), late (0.4 ± 0.04); SCH: early (0.6 ± 0.05), late (0.6 ± 0.05); Kruskal-Wallis test, P = 0.005, Vehicle early vs. SCH early, P = 0.02, Vehicle early vs. SCH late, P = 0.02; Vehicle early vs. late, P = 0.03; SCH early vs. late, P = 0.8). **Alpha** (Vehicle: early (0.4 ± 0.02), late (0.4 ± 0.04); SCH: early (0.6 ± 0.05), late (0.6 ± 0.04); Kruskal-Wallis test, P = 0.005, Vehicle early vs. SCH early, P = 0.3; Vehicle early vs. late, P = 0.4; SCH early vs. late, P = 0.2). **Beta** (Vehicle: early (0.4 ± 0.01), late (0.4 ± 0.02); SCH: early (0.5 ± 0.03), late (0.4 ± 0.03); Kruskal-Wallis test, P = 0.02; Vehicle early vs. late, P = 0.7; SCH early vs. late, P = 0.02). **Low gamma** (Vehicle: early (0.4 ± 0.02), late (0.4 ± 0.03); SCH: early (0.5 ± 0.02), late (0.5 ± 0.02); Kruskal-Wallis test, P = 0.009; Vehicle early vs. late, P = 0.9; SCH early vs. late, P = 0.02). **High gamma** (Vehicle: early (0.4 ± 0.02), late (0.4 ± 0.02); SCH: early (0.5 ± 0.02), late (0.5 ± 0.02); Kruskal-Wallis test, P = 0.05; Vehicle early vs. late, P = 0.4; SCH early vs. late, P = 0.02). (**C**) **mPFC-VTA**. **Theta** (Vehicle: early (0.6 ± 0.04), late (0.6 ± 0.06); SCH: early (0.7 ± 0.03), late (0.7 ± 0.03); Kruskal-Wallis test, P = 0.2; Vehicle early vs. late, P = 0.3; SCH early vs. late, P = 0.2). **Alpha** (Vehicle: early (0.6 ± 0.03), late (0.6 ± 0.03); SCH: early (0.6 ± 0.03), late (0.6 ± 0.03); Kruskal-Wallis test, P = 0.5; Vehicle early vs. late, P = 0.9; SCH early vs. late, P = 0.9). **Beta** (Vehicle: early (0.5 ± 0.02), late (0.5 ± 0.02); SCH: early (0.5 ± 0.02), late (0.5 ± 0.02); Kruskal-Wallis test, P = 0.8; Vehicle early vs. late, P = 0.3; SCH early vs. late, P = 0.05). **Low gamma** (Vehicle: early (0.6 ± 0.02), late (0.6 ± 0.02); SCH: early (0.7 ± 0.03), late (0.6 ± 0.02); Kruskal-Wallis test, P = 0.4; Vehicle early vs. late, P = 0.6; SCH early vs. late, P = 0.02). **High gamma** (Vehicle: early (0.6 ± 0.02), late (0.6 ± 0.03); SCH: early (0.6 ± 0.03), late (0.7 ± 0.03); Kruskal-Wallis test, P = 0.08; Vehicle early vs. late, P = 0.07; SCH early vs. late, P = 0.02). (**D**) **VTA-dHPC**. **Theta** (Vehicle: early (0.4 ± 0.03), late (0.4 ± 0.02); SCH: early (0.5 ± 0.03), late (0.5 ± 0.02); Kruskal-Wallis test, P = 0.05; Vehicle early vs. late, P = 0.9; SCH early vs. late, P = 0.8). **Alpha** (Vehicle: early (0.5 ± 0.01), late (0.4 ± 0.02); SCH: early (0.5 ± 0.03), late (0.5 ± 0.02); Kruskal-Wallis test, P = 0.06; Vehicle early vs. late, P = 0.4; SCH early vs. late, P = 0.2). **Beta** (Vehicle: early (0.4 ± 0.02), late (0.4 ± 0.01); SCH: early (0.4 ± 0.02), late (0.4 ± 0.02); Kruskal-Wallis test, P = 0.04; Vehicle early vs. late, P = 0.05; SCH early vs. late, P = 0.9). **Low gamma** (Vehicle: early (0.3 ± 0.02), late (0.3 ± 0.01); SCH: early (0.4 ± 0.02), late (0.4 ± 0.02); Kruskal-Wallis test, P = 0.2; Vehicle early vs. late, P = 0.2; SCH early vs. late, P = 0.5). **High gamma** (Vehicle: early (0.3 ± 0.01), late (0.3 ± 0.01); SCH: early (0.3 ± 0.01), late (0.4 ± 0.02); Kruskal-Wallis test, P = 0.7; Vehicle early vs. late, P = 0.03; SCH early vs. late, P = 0.08). (**E**) **dHPC-mPFC**. **Theta** (Vehicle: early (0.4 ± 0.02), late (0.4 ± 0.01); SCH: early (0.4 ± 0.03), late (0.4 ± 0.02); Kruskal-Wallis test, P = 0.6; Vehicle early vs. late, P = 0.7; SCH early vs. late, P = 0.8). **Alpha** (Vehicle: early (0.4 ± 0.02), late (0.4 ± 0.02); SCH: early (0.4 ± 0.01), late (0.3 ± 0.009); Kruskal-Wallis test, P = 0.3; Vehicle early vs. late, P = 0.4; SCH early vs. late, P = 0.2). **Beta** (Vehicle: early (0.3 ± 0.01), late (0.3 ± 0.01); SCH: early (0.3 ± 0.005), late (0.3 ± 0.005); Kruskal-Wallis test, P = 0.5; Vehicle early vs. late, P = 0.9; SCH early vs. late, P = 0.4). **Low gamma** (Vehicle: early (0.3 ± 0.01), late (0.3 ± 0.01); SCH: early (0.3 ± 0.01), late (0.3 ± 0.01); Kruskal-Wallis test, P = 0.9; Vehicle early vs. late, P = 0.5; SCH early vs. late, P = 0.02). **High gamma** (Vehicle: early (0.4 ± 0.02), late (0.4 ± 0.02); SCH: early (0.4 ± 0.01), late (0.4 ± 0.02); Kruskal-Wallis test, P = 0.8; Vehicle early vs. late, P = 0.08; SCH early vs. late, P = 0.08). (**F**) **vHPC-dHPC**. **Theta** (Vehicle: early (0.4 ± 0.02), late (0.4 ± 0.03); SCH: early (0.4 ± 0.03), late (0.4 ± 0.02); Kruskal-Wallis test, P = 0.9; Vehicle early vs. late, P = 0.5; SCH early vs. late, P = 0.3). **Alpha** (Vehicle: early (0.4 ± 0.02), late (0.4 ± 0.03); SCH: early (0.4 ± 0.02), late (0.4 ± 0.01); Kruskal-Wallis test, P = 0.05, Vehicle late vs. SCH late, P = 0.03; Vehicle early vs. late, P = 0.1; SCH early vs. late, P = 0.2). **Beta** (Vehicle: early (0.3 ± 0.008), late (0.3 ± 0.007); SCH: early (0.3 ± 0.009), late (0.3 ± 0.009); Kruskal-Wallis test, P = 0.6; Vehicle early vs. late, P = 0.9; SCH early vs. late, P = 0.02). **Low gamma** (Vehicle: early (0.3 ± 0.007), late (0.3 ± 0.007); SCH: early (0.3 ± 0.009), late (0.3 ± 0.007); Kruskal-Wallis test, P = 0.4; Vehicle early vs. late, P = 0.9; SCH early vs. late, P = 0.1). **High gamma** (Vehicle: early (0.3 ± 0.009), late (0.3 ± 0.01); SCH: early (0.3 ± 0.01), late (0.3 ± 0.01); Kruskal-Wallis test, P = 0.8; Vehicle early vs. late, P = 0.2; SCH early vs. late, P = 0.1). Wilcoxon signed-rank tests were used for paired analyses within the Vehicle or SCH group. Kruskal-Wallis test was used for between group analyses. Dunn’s post hoc test was used where applicable. Vehicle (n = 7), SCH (n = 7). * P < 0.05. Group statistics are described as mean ± SEM.
